# Integrated Transcriptomic Analysis Reveals Conserved Inflammatory Transcriptional Signatures Across Bovine Mastitis and Endometritis: Shared Pathways and In Silico Candidate Agents

**DOI:** 10.3390/cimb48101005

**Published:** 2026-09-30

**Authors:** Yueying Zhu, Yonge Feng

**Affiliations:** College of Science, Inner Mongolia Agricultural University, Hohhot 010018, China; zhuyy@emails.imau.edu.cn

**Keywords:** dairy cow, mastitis, endometritis, transcriptome integration analysis, NF-κB signaling conserved pathway

## Abstract

Mastitis and endometritis are prevalent infectious diseases in periparturient dairy cows, yet the shared transcriptional programs of the two conditions remain poorly defined. This study aimed to identify conserved inflammatory signatures and their upstream regulators and to explore candidate modulatory agents in silico. Using two public bovine transcriptomic datasets (GSE75379, GSE125964), we screened directionally concordant differentially expressed genes and performed integrated bioinformatic analyses, including functional enrichment, cell-type signatures (ssGSEA), WGCNA module preservation, cis-eQTL validation and regularized machine learning. Key findings were validated in two independent datasets, and candidate agents were screened using DGIdb 5.0 and HERB 2.0. We identified 383 concordantly upregulated and 61 concordantly downregulated genes, with NF-κB-mediated signaling as the key conserved pathway. The neutrophil signature was the only immune signature consistently enriched in both datasets. *ISG15*, *SYK*, and *SELL* formed a conserved 3-gene core signature with 100% directional concordance across two independent validation cohorts. In silico screening identified the SYK inhibitor fostamatinib and four traditional Chinese medicines as candidate modulators. In conclusion, the NF-κB inflammatory axis and the *ISG15*-*SYK*-*SELL* module form a conserved cross-tissue signature of bacterial mucosal inflammation, offering candidate targets and exploratory reference for the concurrent management of mastitis and endometritis.

## 1. Introduction

The periparturient period is the phase of greatest physiological stress and metabolic burden in dairy cows. Cows around parturition often undergo physiological immunosuppression: neutrophil and lymphocyte function is impaired near calving, and susceptibility to pathogenic infection rises as a result [1,2,3]. Mastitis and endometritis are two major infectious diseases of this period, and both arise at mucosal barriers challenged by bacteria. *Escherichia coli* is a leading cause of acute clinical mastitis; its lipopolysaccharide is sensed through Toll-like receptor 4 and drives a rapid innate inflammatory response in which neutrophils act as the principal effector cells [4]. Bacterial contamination of the postpartum uterus is likewise ubiquitous, with early *Escherichia coli* colonization followed by *Trueperella pyogenes* and anaerobic species, and a substantial proportion of cows develop clinical endometritis, a localized mucosal inflammation recognized clinically by purulent vaginal discharge; endometrial cells sense bacterial pathogen-associated molecular patterns through Toll-like receptors and recruit neutrophils and macrophages [5,6].

Both conditions impose substantial and overlapping costs, reducing milk yield, reproductive performance, and animal longevity. They are also epidemiologically linked, since uterine disease and mastitis form a single periparturient disease network [7], and genetic studies have tied polymorphisms such as *CXCR1* to clinical mastitis risk through shared immune regulatory pathways [8]. Using a farm cohort, Purba et al. [9] found that endometritis was significantly associated with mastitis incidence, as were ovarian follicular cysts, providing direct epidemiological evidence that the two conditions co-occur. This evidence, however, still sits at the phenotypic and genetic-predisposition level.

To our knowledge, no study has systematically tested whether mastitis and endometritis share conserved transcriptional programs and gene modules between the two anatomically distinct tissues. Because the mammary gland and uterus are both mucosal barriers that detect bacteria through pattern-recognition receptors, a conserved cross-tissue inflammatory signature is biologically plausible but remains undefined. We therefore analyzed transcriptomic datasets of *Escherichia coli*-induced mastitis (GSE75379) and clinical endometritis (GSE125964). We compared the two diseases for shared molecular features and upstream regulators, identified cross-tissue conserved core gene modules, and validated them in independent cohorts and genetic databases. We then screened candidate modulatory agents. Our aim was to define a conserved cross-tissue inflammatory signature and a focused core gene module, providing a molecular basis for the concurrent management of mastitis and endometritis in periparturient dairy cows.

## 2. Materials and Methods

### 2.1. Data Processing and Differential Expression Analysis

Two public transcriptomic datasets were downloaded from the Gene Expression Omnibus (GEO) database (https://www.ncbi.nlm.nih.gov/geo/, accessed on 1 July 2026). GSE75379 contains mammary gland transcriptomes of periparturient cows sampled 24 h after *E. coli* infection (6 healthy controls and 6 mastitis cases). GSE125964 contains endometrial epithelial transcriptomes collected on postpartum day 7 (D7), from which we used the clinical endometritis subgroup (10 healthy controls and 10 clinical endometritis cases). The two datasets were analyzed independently throughout this study. For GSE75379, raw read counts were retained, genes with a count > 10 in fewer than three samples were filtered out, and differentially expressed genes (DEGs) were identified using DESeq2 (version 1.50.0) [10] under a paired design controlling for individual cow (model = ~cow + group), with the Wald test and Benjamini–Hochberg correction. For GSE125964, differential statistics were not recalculated because the original study had already analyzed raw read counts via DESeq2 (version 1.18.1). Detailed characteristics of all discovery and validation cohorts, including sequencing platform, breed, parity, sample size, and differential-expression criteria, are summarized in Supplementary Table S1. We applied consistent thresholds to the reported statistics after reorienting all contrasts to disease versus control. Accordingly, DEGs across both datasets were defined using an adjusted *p* < 0.05 and |log2FC| > 1, generating distinct up-and down-regulated genes sets for subsequent concordance and intersection analysis. Transformed continuous expression matrices were used only for visualization and the downstream ssGSEA, WGCNA, and machine-learning analyses, never for differential-expression calling.

### 2.2. Gene Set Enrichment Analysis (GSEA)

Gene set enrichment analysis (GSEA) [11] was performed separately for the two datasets using the clusterProfiler (version 4.18.4) R package, with bovine GO Biological Process terms from org.Bt.eg.db as reference gene sets. All genes were ranked by descending log2FC, with a padj cutoff of 0.1, minimum gene set size of 10, and maximum gene set size of 500. Significantly enriched pathways (adjusted *p* < 0.05) shared by both diseases were intersected and classified by the direction of normalized enrichment scores. Semantic redundancy reduction of concordant pathways was conducted using the simplify function in clusterProfiler with GOSemSim (version 2.36.0) at a similarity cutoff of 0.7.

### 2.3. Cell-Type Enrichment Analysis Based on a Single-Cell Expression Atlas

The relative enrichment of each cell-type marker signature in each sample was evaluated using the single-sample gene set enrichment analysis (ssGSEA) algorithm. Cell-type marker genes were derived from a bovine multi-tissue single-cell expression atlas [12]. Marker gene lists for each cell type were obtained from the supplementary tables of the publication, and cell types annotated to the mammary gland and intima of the corpus uteri were selected. For each dataset, we intersected the marker genes with the genes detected in that dataset and kept only cell types with at least three intersecting genes, so that the signature gene sets remained representative and stable in our data. We then scored each sample using the ssgsea function in the GSVA package (version 2.6.2) [13] with a Gaussian kernel, which gave a sample-by-cell-type signature enrichment score matrix; these scores reflect the relative coordinate enrichment of marker signatures rather than estimates of absolute cell proportions. For both datasets, the ssGSEA activity scores of each cell type were compared between the disease and control groups using a two-sided Wilcoxon rank-sum test. *p* values were adjusted by the Benjamini–Hochberg method [14], and FDR < 0.05 was considered significant. The direction and magnitude of changes in cell-type signature enrichment were quantified as the difference in mean ssGSEA scores between the two groups (Supplementary Table S5).

### 2.4. Screening of Concordant Shared Intersection Genes and Hub Genes

The directionally concordant intersection of differentially expressed genes (DEGs) was extracted from the two datasets, with genes showing opposite expression changes excluded. A protein–protein interaction (PPI) network was constructed using the STRING database (https://cn.string-db.org/, accessed on 3 July 2026) [15] at a confidence score cutoff of ≥0.7, and visualized in Cytoscape 3.10.4 [16]. For the co-upregulated network, only genes with degree > 3 were retained, and the top 30 hub genes were identified via the MCC algorithm [17]. For the co-downregulated network, isolated genes and two-node micro-modules were removed to retain the core module, yielding the final core set of concordant shared genes. GO/KEGG enrichment analysis was performed on these genes using the clusterProfiler R package [18] with an FDR threshold of <0.05.

### 2.5. Immune–Lipid Metabolism Coupling Analysis

To determine whether downregulated shared genes arose from tissue cellular compositional shifts or cell-intrinsic metabolic reprogramming, three ssGSEA gene sets were constructed: upregulated core genes, downregulated core genes, and myeloid cell-type signature markers. Based on sample-level ssGSEA scores, Spearman’s simple correlation was calculated between the immune activation score (upregulated core genes) and the fatty acid oxidation score (metabolism-enriched downregulated core genes). Spearman’s partial correlation was then computed with myeloid cell-type signature score as a covariate to control for cellular composition confounding. A significantly negative simple correlation paired with a non-significant partial correlation would indicate the negative association is driven primarily by cellular compositional changes.

### 2.6. Transcription Factor Activity Inference and Promoter Binding Motif Analysis

Transcription factor (TF) activity was inferred using the viper R package (version 1.44.0) [19] with the DoRothEA v2 regulatory network [20], retaining only confidence A/B interactions. Case–control *t*-tests with Benjamini–Hochberg correction identified shared differentially active TFs (adjusted *p* < 0.05 in both diseases). For binding motif validation, promoter sequences (2000 bp upstream to 200 bp downstream of the transcription start site) were extracted from the bovine reference genome (bosTau9/ARS-UCD1.2, the bovine reference assembly used throughout) and scanned with JASPAR 2022 position weight matrices [21] via the TFBSTools R package (version 1.48.0) [22] at a 95% relative score threshold. As bovine-specific curated matrices were limited, we used the corresponding curated vertebrate profiles for the three a priori candidate TFs. This cross-species motif transfer is supported by the evolutionary conservation of TF DNA-binding specificity among mammals. Motif scanning was interpreted as in silico evidence of potential promoter occupancy rather than direct evidence of TF binding. Fisher’s exact test assessed motif enrichment against commonly expressed background genes, with odds ratios and 95% CIs reported. Analyses were run on both co-upregulated and core shared genes; *p*-values for the three a priori candidate TFs were BH adjusted, with unadjusted values also provided.

### 2.7. WGCNA and Cross-Dataset Module Preservation Analysis

A weighted gene co-expression network was constructed with WGCNA [23] on the harmonized log2(CPM + 1), cross-dataset quantile-normalized matrix described in Section 2.1, using GSE75379 as the reference network and GSE125964 as the test network. Cross-dataset module preservation was assessed using the modulePreservation() function of WGCNA [24] with 200 permutations, and the Z-summary statistic was calculated. Because the sample size of the mastitis reference network (*n* = 12) is below the minimum generally recommended for this method, the random control module (gold, constructed by random sampling from the whole network as the noise baseline of the permutation test) tended to show inflated Z-summary values. Therefore, instead of applying the fixed threshold (Z ≥ 10), we used the Z-summary of the gold module as the noise baseline and considered only modules that markedly exceeded this baseline as having strong evidence of preservation. The enrichment of concordant shared genes in each module was evaluated with a hypergeometric test (*p* < 0.05), and genes with a module membership (kME) > 0.7 were defined as module core genes.

### 2.8. Validation of Genetic Evidence from cis-eQTLs

Gene symbols were mapped to ENSBTAG identifiers using the CattleGTEx database (https://cattlegtex.kiz.ac.cn/, accessed on 8 July 2026) [25]. Gene-level ACAT-aggregated cis-eQTL *p* values were retrieved and processed with the data.table (version 1.18.0) and dplyr (version 1.1.4) R packages. Significance was defined as a BH-adjusted q value < 0.05. Analyses covered mammary gland and uterus, and were extended to immune-related tissues (blood, lymph node, spleen) for validation.

### 2.9. Cross-Disease Transferability Analysis and Resampling-Based Stability Assessment

Genes with significant cis-eQTL evidence from the upstream integrative cross-disease analysis were used as a biologically motivated candidate feature pool. Regularized logistic regression models were developed using the mastitis dataset GSE75379 only. Because GSE125964 was used for the upstream identification of shared disease features, it was not treated as a fully independent external test set in this analysis but solely as a cross-disease transfer cohort. Expression values were standardized using the mean and standard deviation estimated from GSE75379, with identical parameters applied to GSE125964. Ridge (α = 0), LASSO (α = 1), and ElasticNet (α = 0.5) logistic regression models were fitted using glmnet (version 4.1.10) [26], with the regularization parameter λ selected by five-fold cross-validation within GSE75379. Cross-disease transferability was assessed in GSE125964 using ROC-AUC and 95% confidence intervals. Model-derived feature prioritization was performed exclusively in GSE75379: consensus candidates were defined as genes simultaneously ranked among the top 10 by training-set SHAP importance in both Ridge and ElasticNet and assigned non-zero coefficients by LASSO. SHAP values were calculated with fastshap (version 0.1.1) [27] using training-set samples only. To assess feature-selection stability, 1000 stratified bootstrap resamples were generated from GSE75379, in which controls and cases were resampled separately with replacement to retain the original 6/6 class sizes. Within each resample, standardization, three-fold cross-validation, model fitting, and consensus selection were repeated, and selection frequency and coefficient-direction stability were summarized across resamples.

### 2.10. Validation in Independent Datasets

Two bovine databases were used for gene- and pathway-level cross-validation. The mastitis database (GSE24217, 9 controls, 12 cases) was obtained from GEO, comprising mammary gland tissues collected 24 h after *E. coli* challenge in cows at 4–6 weeks postpartum. The endometritis database (12 controls, 6 cases) was derived from a published periparturient transcriptomic study [28], only summarized DEG tables were available without individual expression data, so it was used solely for expression trend consistency and pathway over-representation analysis. To incorporate expression direction, each two-sided *p* was converted to a one-sided Z score in the direction expected from the discovery data, assigned a positive sign when the validation and discovery fold changes agreed and a negative sign when they opposed each other, and then combined across available genes using Stouffer’s method [29], and the directional concordance rate was calculated. At the pathway level, the TNF and NF-κB signaling pathways were selected. For the mastitis cohort, KEGG pathway genes were scored per sample via ssGSEA, and groups were compared by independent-samples t-test. For the endometritis cohort, pathway genes were converted to Ensembl IDs via the biomaRt R package (version 2.66.2) [30]. Fisher’s exact test was applied for over-representation analysis of pathway genes within the significantly upregulated gene set (*p* < 0.05, log2FC > 0), with corresponding odds ratios calculated.

### 2.11. Correlation and Co-Expression Analysis of Core Genes and Immune Cells

Within the final gene module, associations of module genes with immune cell populations and among themselves were examined separately in each dataset. Cell-type signature enrichment scores obtained by ssGSEA were correlated with module-gene expression by Pearson correlation, with *p* values Benjamini–Hochberg adjusted across the gene–cell-type matrix and significance set at adjusted *p* < 0.05 (visualized as a heatmap). Pairwise co-expression among the three module genes was computed on transformed expression values by Pearson correlation coefficients, with significance from two-sided *t*-tests (*p* < 0.05), and was rendered as a co-expression network whose edge width and color encode the correlation coefficient.

### 2.12. Screening of Candidate Modulatory Agents

Candidate modulatory agents for the three module genes were explored in silico with two complementary databases. Drug–gene interactions were retrieved from DGIdb 5.0 (https://beta.dgidb.org/, accessed on 25 July 2026) [31] and restricted to formally approved agents with a drug–gene interaction score ≥ 0.5. Traditional Chinese medicine–gene relationships were obtained from HERB 2.0 (http://47.92.70.12/, accessed on 25 July 2026) [32], retaining only relationships supported by published experimental or literature evidence and excluding inferred, prediction-only associations. All retrieved agents are reported as exploratory in silico candidates for hypothesis generation, not as evidence of therapeutic efficacy.

## 3. Results

### 3.1. Differential Expression of the Two Diseases and Shared Differentially Expressed Genes

Following the predefined significance thresholds, differentially expressed gene sets were identified for mastitis (GSE75379) and endometritis (GSE125964), respectively (Figure 1a–d). At the predefined thresholds, 1924 DEGs (1231 upregulated, 693 downregulated) were identified in GSE75379 and 1296 DEGs (748 upregulated, 548 downregulated) in GSE125964. The directionally concordant intersection was extracted by excluding genes with opposite expression changes, yielding 383 co-upregulated genes and 61 co-downregulated genes (Figure 1e). The complete list of the 444 directionally concordant shared genes, together with their per-dataset statistics and membership in the downstream core set, is provided in Supplementary Table S2. The markedly larger number of co-upregulated genes relative to co-downregulated genes indicates that shared molecular features of the two diseases are predominantly characterized by immune and inflammatory activation, whereas common downregulated signatures are comparatively limited.

### 3.2. GSEA Reveals Shared Activation of Immune and Inflammatory Pathways in the Two Diseases

As shown in Figure 2a, significantly enriched GSEA pathways in mastitis were predominantly concentrated in the upregulated gene set. T cell activation and leukocyte cell–cell adhesion showed the highest enrichment significance, with defense response, response to external biotic stimulus, response to biotic stimulus, chemotaxis, and regulation of lymphocyte activation also prominently enriched, presenting a typical anti-infective immune activation profile. In endometritis, most pathways were upregulated while only a few were significantly downregulated (Figure 2b). The most prominent feature was the marked downregulation of cytokine-mediated signaling pathways. The upregulated immune defense pathways largely overlapped with those in mastitis, and three additional endometritis-specific pathway categories were identified: cilium assembly, cilium organization, and plasma membrane cell projection assembly. Following semantic redundancy reduction, the intersection of shared GSEA pathways between the two diseases (Figure 2c) demonstrated that the vast majority of immune and inflammatory pathways were concordantly upregulated in both conditions. Significant enrichment spanned the complete cascade from pathogen sensing and cytokine signaling to immune cell activation and defensive clearance, indicating closely concordant immune mechanisms underlying periparturient infections in the two organs. Meanwhile, clear tissue-specific differences were observed. Endometritis was characterized by downregulated epithelial cytoskeleton-related pathways and upregulated lipid metabolism- and virus-related pathways. By contrast, mastitis exhibited more prominent innate immune effector pathways (e.g., phagocytosis, antimicrobial peptides) along with stronger negative immune regulation.

### 3.3. Cell-Type Signature Enrichment in the Two Diseases

ssGSEA scoring based on bovine tissue-specific cell type markers (Figure 3) revealed that in the mastitis dataset, neutrophils (delta = 0.441, FDR = 0.014), macrophages (delta = 0.222, FDR = 0.014), and other immune cells (delta = 0.312, FDR = 0.014) were significantly increased in the infected group, consistent with a predominant early innate immune response at this stage. In the endometritis dataset, nine cell-type signatures showed significant elevation. Beyond innate populations such as neutrophils (delta = 0.257, FDR = 0.026), the most prominent feature was the strong enrichment of adaptive and cytotoxic immune-cell signatures, including cytotoxic CD8+ T cells, NKT cells, CD4+ T cells, T cells, and NK cells, accompanied by significant changes in blood and lymphatic endothelial signatures. The macrophage signature showed an increasing trend but did not reach significance (FDR = 0.058). Overall, the altered signatures in mastitis clustered within innate immunity and myeloid lineages, whereas endometritis was characterized by adaptive and cytotoxic immunity. The neutrophil signature was the only one significantly enriched in both datasets.

### 3.4. Screening of Core Shared Genes by PPI Network Analysis

Analysis of the PPI network of co-upregulated genes (degree > 3) and the top 30 MCC hub genes (Figure 4a,b) showed that upregulated genes formed innate immune activation hubs centered on NF-κB/JAK-STAT signaling, with *IL1B*, *IL6*, *TLR2*, *CCL5*, and *CXCL*8 occupying central positions in the network. In the co-downregulated gene network (Figure 4c), a peroxisomal fatty acid β-oxidation module centered on *EHHADH*, *ACSS2*, and *ACOX2* was downregulated, consistent with previous reports on inflammation–metabolism interplay [33,34]. GO/KEGG enrichment analysis of the 47 core genes (30 upregulated and 17 downregulated) showed that upregulated genes were enriched in inflammatory response, defense response to bacterium, and cytokine–cytokine receptor interaction (Figure 4d,e), whereas downregulated genes were enriched in fatty acid oxidation and peroxisome metabolism (Figure 4f,g) (The stepwise WGCNA, cis-eQTL, and machine-learning annotation of each core gene is provided in Supplementary Table S3).

### 3.5. Immune–Lipid Metabolism Coupling

The ssGSEA-derived immune activation score and fatty acid oxidation score exhibited a significant negative correlation in both datasets (mastitis: rho = −0.90, *p* < 0.001; endometritis: rho = −0.53, *p* = 0.017) (Figure 5a,b). However, when myeloid cell-type signature score was included as a covariate in Spearman’s partial correlation analysis, this negative correlation lost statistical significance in both datasets (mastitis: rho = −0.10, *p* = 0.759; endometritis: rho = −0.23, *p* = 0.349). The apparent negative association between immune activation and suppressed fatty acid oxidation thus appears to be attributable mainly to shifts in myeloid cell-type composition during inflammation rather than to cell-intrinsic metabolic reprogramming of tissue-resident cells.

### 3.6. Analysis of Upstream Transcription Factor Regulation

VIPER-based TF activity inference revealed that *HIF1A* and *RARA* exhibited significantly increased activity in both diseases. Activity differences for *HIF1A* were 3.70 in mastitis and 4.93 in endometritis, while those for *RARA* were 3.06 and 1.73, respectively (all adjusted *p* < 0.05). Based on these findings, three TFs were selected for binding motif validation: *HIF1A*, the TF with the most consistently elevated activity across both diseases identified by VIPER, and *RELA* and *NFKB1*, core subunits of the NF-κB/TNF pathway prominently enriched among core shared genes [35].

Promoter binding motif enrichment was evaluated in two sequentially refined gene sets. In the set of 383 concordantly upregulated genes, 346 had retrievable bosTau9 promoter sequences, as did 46 of the 47 core genes. In the 383-gene set (Figure 6a), both NF-κB family motifs were significantly enriched after BH correction: RELA occurred in 17.3% of genes versus 12.9% of the 8281-gene background (OR = 1.41, 95% CI 1.04–1.88), and NFKB1 in 1.7% versus 0.5% (OR = 3.38, 1.17–8.05), both at adjusted *p* = 0.023, whereas the HIF1A motif was significantly depleted (22.0% vs. 27.6%, OR = 0.74, adjusted *p* = 0.023). In the more focused set of 47 core shared genes (Figure 6b), the RELA occurrence rate was numerically higher (23.9% vs. 13.1% background, OR = 2.09) with a nominal unadjusted *p* = 0.045, but this did not survive BH correction (adjusted *p* = 0.135) and its confidence interval crossed unity (0.95–4.22); NFKB1 had no occurrence in this set, and HIF1A showed only a non-significant downward trend (17.4% vs. 27.4%, OR = 0.56). Canonical NF-κB targets including IL1B, IL6, and CXCL8 nevertheless carried RELA/NFKB1 motifs. Motif enrichment thus received statistical support at the broad shared-gene level and directional support at the focused core-gene level, consistent with NF-κB acting as a shared upstream transcriptional regulatory program in both diseases.

### 3.7. Cross-Tissue Module Preservation by WGCNA

A total of 24 co-expression modules were identified in the mastitis reference network (Figure 7a,b). As shown in Figure 7c, the blue module achieved a Z-summary of 28.7, markedly exceeding that of the gold random control module (Z-summary = 10.65). Consistent results were obtained from the independent evaluation based on median rank (Figure 7d): the blue module ranked first by Z-summary and fourth by median rank (where a smaller value denotes stronger preservation), together indicating the most robust cross-tissue preservation. The blue module was strongly positively correlated with mastitis disease status (r = 0.94, *p* = 4 × 10^−6^) and harbored 27 of the 47 core shared genes, among which 22 genes had a module membership (kME) > 0.7: *IL1RN*, *IL10*, *SOCS3*, *IL1B*, *LYN*, *SYK*, *ISG15*, *NFKBIA*, *RELB*, *JAK2*, *CD40*, *IL6R*, *CXCL8*, *TLR2*, *CD80*, *ICAM1*, *SELL*, *TNFAIP3*, *CCL20*, *IL6*, *IRF1*, and *CD69* (Figure 7e; Supplementary Table S4).

### 3.8. Genetic Evidence from cis-eQTLs

Thirteen of the 22 blue module core genes exhibited significant cis-eQTL signals (BH-adjusted q < 0.05) in at least one tissue (Figure 8). *IL6R* was the only gene reaching statistical significance in both the mammary gland and the uterus, with an exceptionally strong cis-eQTL signal in the uterus (*p* = 6.01 × 10^−17^, q = 1.34 × 10^−12^, β = −0.18), representing the most significant hit among the 13 validated genes. *IL6* was significant in both the mammary gland (*p* = 1.25 × 10^−5^, β = 0.29) and immune tissues (*p* = 4.67 × 10^−6^, β = 0.39), and *SYK* likewise showed significant signals in the mammary gland (*p* = 1.04 × 10^−6^) and immune tissues (*p* = 5.60 × 10^−9^). Regarding effect directions, most genes exerted positive regulatory effects (β > 0). *NFKBIA* and *ICAM1* exhibited negative effects in the mammary gland (β = −0.11 and −0.18, respectively), while *TLR2* and *IL6R* also showed negative effects in the uterus (β = −0.17 and −0.18, respectively).

### 3.9. Cross-Disease Transferability and Bootstrap Stability

Using the 13 cis-eQTL-supported candidate genes, Ridge yielded the highest cross-disease transfer AUC in GSE125964 (AUC = 0.92, 95% CI 0.797–1.000), followed by ElasticNet (AUC = 0.90, 95% CI 0.760–1.000) and LASSO (AUC = 0.84, 95% CI 0.659–1.000) (Figure 9a) (Supplementary Table S6c). Given the small training cohort and wide confidence intervals, these results are interpreted as exploratory evidence of cross-disease transferability rather than definitive external predictive performance. Training-set-only consensus selection identified four raw candidates: ISG15, SELL, SYK, and IL6R. In 1000 stratified bootstrap resamples (all valid), each candidate retained a positive coefficient whenever selected; their three-algorithm consensus frequencies were 91.3% for ISG15, 74.4% for SELL, 63.0% for SYK, and 53.5% for IL6R (Supplementary Table S6a,b). Model interpretability was assessed using SHAP values derived solely from the training set, computed exclusively on GSE75379 with no information from the transfer cohort. The four raw consensus candidates ranked among the leading features across the three models and together constituted the entire set of non-zero LASSO coefficients. At the individual-sample level, however, each candidate displayed both positive and negative SHAP values straddling zero (Figure 9b–d), indicating that their influence on the predicted disease probability was not consistently in one direction but varied across animals.

### 3.10. Validation in Independent Datasets

Independent validation was first performed for all four raw machine-learning candidates. As shown in Figure 10a, in the mastitis dataset GSE24217, all four raw candidates were significantly upregulated in infected cows and concordant with their discovery directions (four-gene direction-aware combined Z = 10.27, *p* < 0.001). The independent endometrial summary dataset contained three of the four candidates: ISG15 (log_2_FC = 2.164, *p* = 0.0199) and SELL (log_2_FC = 3.326, *p* = 2.44 × 10^−7^) were significantly upregulated, but IL6R was significantly downregulated (log_2_FC = −1.214, *p* = 0.0147), reversing both its discovery direction and its direction in the mastitis validation set, while SYK was absent from the summary table. IL6R was supported by cross-tissue cis-eQTL evidence and stable resampling selection, but its direction reversal in endometritis meant that it failed to satisfy cross-disease directional conservation. It was therefore retained only as a supplementary candidate and excluded from the focused cross-tissue module. By contrast, the concordant upregulation of SYK in GSE24217 supported its retention in the module. The focused module was consequently defined as ISG15, SYK and SELL. For this final module, the direction-aware combined Z was 8.73 (*p* < 0.001) with 100% directional concordance in GSE24217; in the endometrial dataset, the two detectable module genes (ISG15, SELL) gave Z = 5.30 (*p* = 5.9 × 10^−8^), again with 100% concordance, compared with Z = 2.92 (*p* = 0.00177) when the discordant IL6R was retained. Per-gene log2 fold changes, *p* values, and direction-aware Stouffer statistics for all four raw candidates in both independent cohorts are provided in Supplementary Table S7a,b.

At the pathway level, TNF and NF-κB pathway activity was significantly higher in the GSE24217 mastitis cohort (TNF: t = −11.819, *p* = 2.78 × 10^−9^; NF-κB: t = −15.083, *p* = 3.46 × 10^−10^). In the independent endometritis cohort, TNF signaling was enriched among upregulated genes (28/29 pathway genes; Fisher’s *p* = 3.66 × 10^−4^; OR = 13.17), while all 24 detected NF-κB pathway genes were upregulated (Fisher’s *p* = 1.79 × 10^−4^). These results support conserved activation of the TNF/NF-κB inflammatory axis across independent cohorts.

### 3.11. Correlation and Co-Expression Network of Core Genes with Immune Cells

Pearson correlation analysis between the three core genes and cell-type ssGSEA scores (Figure 11a) revealed that *ISG15*, *SYK*, and *SELL* were consistently positively correlated with immune cell scores in both diseases, but their correlation spectra differed markedly between tissues. In mastitis, significant correlations were concentrated in immune cell populations: *ISG15* and *SELL* were both significantly positively correlated with neutrophils, macrophages, other immune cells, and CD4+ T cells; *SYK* was significantly positively correlated with neutrophils and other immune cells. No significant correlations were observed for non-immune cells. In endometritis, correlations were more widespread. *SYK* was the most prominent, showing significant positive correlations with multiple immune cell types as well as glial cells, smooth muscle cells, fibroblasts, and stromal cells of the uterine intima, and a significant negative correlation only with luminal cells. *ISG15* was significantly correlated with multiple cytotoxic and lymphoid cell types, and *SELL* was mainly associated with neutrophils and T cells. The broad positive correlations of the three genes with immune cells suggest that they may jointly participate in immune cell recruitment and activation under inflammatory conditions, and the tissue-specific differences in their correlation profiles are consistent with the distinct cellular composition characteristics of the two diseases.

Pearson co-expression analysis showed that the three genes were significantly positively correlated with one another in both datasets (Figure 11b), indicating that these three genes form a stable and conserved co-expression module across mucosal bacterial infections at different anatomical sites, and may jointly mediate immune cell recruitment and coordinated activation of inflammatory signaling following infection.

### 3.12. In Silico Screening of Candidate Modulatory Agents

Candidate modulatory for the three core genes were explored in silico using DGIdb 5.0 and HERB 2.0 (Table 1). For Western drugs retrieved from DGIdb, SYK was linked to fostamatinib and its disodium salt, a SYK inhibitor FDA-approved for immune thrombocytopenia that acts on a kinase upstream of NF-κB signaling. *ISG15* was only associated with the chemotherapeutic agent irinotecan hydrochloride, which carries no inflammation-related indications, and no approved agents were identified for *SELL*. No single approved Western drug covered the whole shared module, and constraints such as lactational milk residues and mismatched indications limit their direct translation to periparturient dairy cows.

Based on experimentally validated evidence from the HERB database, four traditional Chinese medicine candidates supported by published experimental evidence were identified. *Dictamni Cortex* and toxicity-attenuated processed *Aconiti Radix* (*Aconiti Radix Cocta*) have been reported to inhibit SYK-mediated inflammatory signaling and reduce the release of pro-inflammatory cytokines including IL-6 and TNF-α [36,37]. The classic tonifying and defensive formula Yu–Ping–Feng San activates interferon-stimulated response elements in macrophages and induces the expression of antiviral proteins such as *ISG15*, supporting its role in modulating early interferon signaling and enhancing anti-infective defense [38]. Root extracts of *Ononis spinosa* downregulate the adhesion molecules *CD11b*/*CD18*, preserve *CD62L* (*SELL*) expression, and inhibit IL-8 and TNF-α release in LPS-stimulated neutrophils, indicating potential to attenuate leukocyte adhesion and migratory activation and reduce immune cell infiltration at inflammatory sites [39]. The four candidates correspond to three complementary stages of the mucosal inflammatory cascade: innate immune regulation, inflammatory signal transduction, and leukocyte migration. These database-derived candidates are in silico leads requiring experimental validation, not evidence of therapeutic efficacy. The complete DGIdb retrieval records, including non-approved and below-threshold interactions together with the filtering flags, are provided in Supplementary Table S8a, and the retained literature-supported herbal candidates are listed in Supplementary Table S8b.

## 4. Discussion

Mastitis and endometritis are highly prevalent infectious diseases in periparturient dairy cows. Epidemiological evidence has shown that the occurrence of one disease significantly elevates the risk of the other [9], yet the molecular basis underlying their cross-organ co-occurrence remains incompletely characterized. In this study, we integrated transcriptomic datasets of the two diseases and performed stepwise analyses spanning functional pathways, cell-type signatures, core gene modules, and upstream regulatory mechanisms, showing that both diseases share concordant anti-infective immune activation signatures.

GSEA results revealed extensive overlap in significantly enriched pathways between the two diseases, spanning the complete response cascade from pathogen sensing and cytokine signaling to immune cell activation and effector clearance, indicating concordant immune mechanisms underlying infectious inflammation in the two organs. Nevertheless, clear tissue-specific features were observed. Endometritis was characterized by downregulated cytokine signaling and impaired epithelial cytoskeleton pathways, whereas mastitis exhibited more prominent innate immune effector pathways including phagocytosis and antimicrobial peptides, a distinction consistent with subsequent immune cell composition analysis. ssGSEA of tissue-specific cell type markers showed that mastitis was dominated by myeloid cell-type signatures (neutrophils and macrophages), whereas nine cell-type signatures were significantly enriched in endometritis, most notably adaptive and cytotoxic immune cell populations. The neutrophil signature was the only one consistently enriched in both datasets, aligning with the shared anti-infective program and suggesting they serve as a common effector cell population across both conditions. However, the divergent immune cell repertoires may stem from differences in disease stage at sample collection, or reflect inherent disparities in tissue microenvironment and pathogen spectra, which cannot be fully disentangled with the current data.

Following directionally concordant intersection of differentially expressed genes, 383 co-upregulated and 61 co-downregulated genes were identified. These were further refined to 47 core shared genes via PPI network construction and MCC topological hub screening. The 17 downregulated core genes were enriched in metabolic functions including fatty acid oxidation, initially suggestive of metabolic reprogramming in the shared signature. However, the negative correlation between the immune activation score and fatty acid oxidation score lost significance after adjusting for the myeloid cell-type signature score, indicating that the observed metabolic suppression signal primarily arises from altered proportions of infiltrating immune cells rather than cell-intrinsic metabolic reprogramming of tissue-resident cells. This suggests that the shared features predominantly reflect inflammatory activation rather than widespread metabolic remodeling. Independent metabolomic studies of periparturient uterine diseases have also reported significant alterations in lipid metabolism-related molecules [40,41], consistent with our transcriptomic observations, but the cellular origin of these metabolic changes similarly requires further clarification.

Weighted gene co-expression network analysis (WGCNA) further corroborated the functional coordination of core genes. The blue module harbored 27 of the 47 core shared genes, 22 of which had a module membership (kME) value above 0.7 and served as intramodular hubs. Subsequent integration with CattleGTEx cis-eQTL data revealed significant cis-eQTL signals for 13 of the 22 hub genes. Notably, IL6R was the only hub gene with significant cis-eQTL associations in both mammary gland and uterus, suggesting partial modulation of the module by host genetic background; nevertheless, IL6R was not retained in the final three-gene module because its expression direction was discordant in the independent endometritis cohort.

Upstream regulatory analysis delineated candidate upstream transcriptional regulators of the shared disease program. Promoter motif enrichment, VIPER-based TF activity inference, and external dataset validation converged on the NF-κB inflammatory axis as a shared upstream regulator. RELA motifs were significantly enriched among the 383 concordantly upregulated genes (Benjamini–Hochberg-adjusted *p* < 0.05); a directionally consistent enrichment was seen in the 47-gene core set but remained nominal after multiple-testing correction at this smaller set size. Both the mammary gland and uterus are mucosal barrier tissues that detect bacterial pathogens via pattern recognition receptors, and NF-κB activation downstream of Toll-like receptors represents a conserved rapid response pathway [42,43]. This sensing-transcription-effector cascade may constitute a shared molecular feature of infection in both organs. By contrast, *HIF1A* exhibited a dissociation between activity and DNA binding: its regulon activity was elevated but direct proximal promoter binding was scarce, suggesting that hypoxia–inflammation crosstalk may contribute to the shared inflammatory program via indirect mechanisms, consistent with previously reported interactions between *HIF1A* and the NF-κB pathway [44]. Collectively, these findings indicate that the NF-κB inflammatory axis may represent a candidate upstream node for concurrent modulation of inflammation in both diseases.

Three regularized logistic regression models (Ridge, LASSO, and ElasticNet) were trained exclusively on the mastitis dataset and transferred, without refitting, to the endometritis dataset to assess cross-disease transferability. Training-set-only consensus selection yielded four raw candidates, all of which retained positive coefficients with no direction reversal across 1000 stratified bootstrap resamples. Of these, *IL6R* was excluded from the focused module because it was downregulated in the independent endometritis cohort despite its strong cross-tissue cis-eQTL support, leaving *ISG15*, *SYK*, and *SELL* as the final three-gene signature. Because the training cohort was small and the transfer confidence intervals were wide, the reported AUCs should be interpreted as exploratory evidence of cross-tissue transferability rather than as estimates of external predictive performance, and the signature warrants validation in larger independent cohorts.

The biological relevance of the three genes is supported by several convergent lines of evidence. All three are highly connected hub genes with kME > 0.7 within the blue co-expression module, and they exhibit significant positive correlations with one another in both datasets, consistent with a coordinated unit under shared upstream regulation. Functionally, the three genes correspond to interferon response initiation, immune receptor signal transduction, and leukocyte adhesion/migration, respectively, spanning the continuous inflammatory cascade from pathogen sensing to effector cell recruitment. Immune cell correlation analysis further delineated their associations with specific immune cell subsets: *ISG15* and *SELL* were closely aligned with myeloid and T-cell signatures in mastitis, whereas *SYK* correlated with the widest spectrum of immune cells in endometritis and was also associated with certain non-immune cell types, suggesting its expression may also reflect state alterations in tissue-resident cells. Notably, the three genes are proposed as a conserved cross-tissue inflammatory signature rather than as disease-specific diagnostic markers; their causal roles and any modulatory potential still require validation in bovine cellular and animal models.

In summary, this study systematically characterized shared transcriptomic signatures of bovine mastitis and endometritis at the periparturient stage and delineated the scope of their cross-organ molecular similarities. By integrating motif enrichment, TF activity inference, and external cohort validation, our convergent analyses support the NF-κB inflammatory axis as a key shared upstream regulatory program. The stepwise convergent screening framework applied in this study refined the initial DEG list into a convergent candidate set supported by three complementary lines of evidence: expression concordance, network co-regulation, and genetic cis-eQTL support.

This study has several limitations. The discovery cohorts were small and derived from a single platform within a specific postpartum window, and the independent endometritis cohort was available only as summary-level differential-expression results, which precluded individual-level classifier refitting and contributed to the wide transfer confidence intervals. Because bulk tissue RNA-seq was used, ssGSEA reflects cell-type signature enrichment rather than direct measurement of cell abundance. Motif scanning relied on cross-species position-weight matrices and thus provides in silico rather than direct binding evidence, and the candidate agents arose solely from database curation rather than efficacy testing.

## 5. Conclusions

This cross-tissue transcriptomic integration study shows that *E. coli*-induced mastitis and clinical endometritis in periparturient dairy cows share molecular features dominated by anti-infective immune activation, and that the NF-κB-mediated inflammatory axis is strongly implicated as a shared upstream regulatory pathway in both conditions. The *ISG15*-*SYK*-*SELL* module forms a conserved cross-tissue three-gene signature supported by cis-eQTL evidence and independent cohort validation and may serve as candidate marker genes and modulatory nodes for concurrent management of the two conditions. Fostamatinib and four traditional Chinese medicine candidates emerged as in silico candidate modulators of this shared signature, although their efficacy and clinical applicability require experimental validation. Collectively, this study offers an exploratory molecular reference for the concurrent management of mastitis and endometritis in periparturient dairy cows.

## Figures and Tables

**Figure 1:**
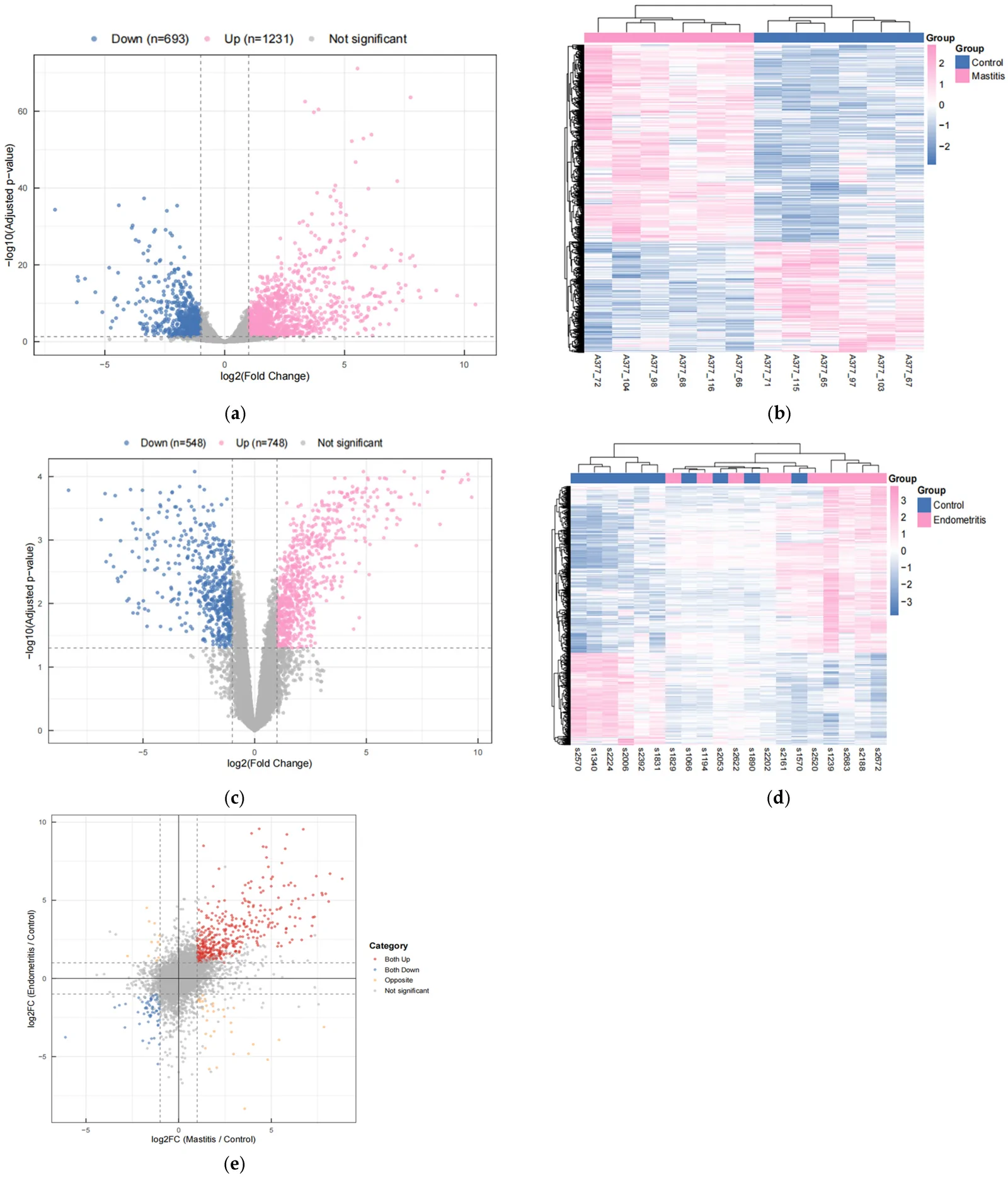
Differential expression profiles of the two diseases and screening of shared differentially expressed genes. (**a**) Volcano plot of the mastitis dataset GSE75379. (**b**) Heatmap of the mastitis dataset GSE75379. (**c**) Volcano plot of the endometritis dataset GSE125964; (**d**) Heatmap of the endometritis dataset GSE125964. (**e**) Four-quadrant plot of the directional concordance of differentially expressed genes between the two diseases.

**Figure 2:**
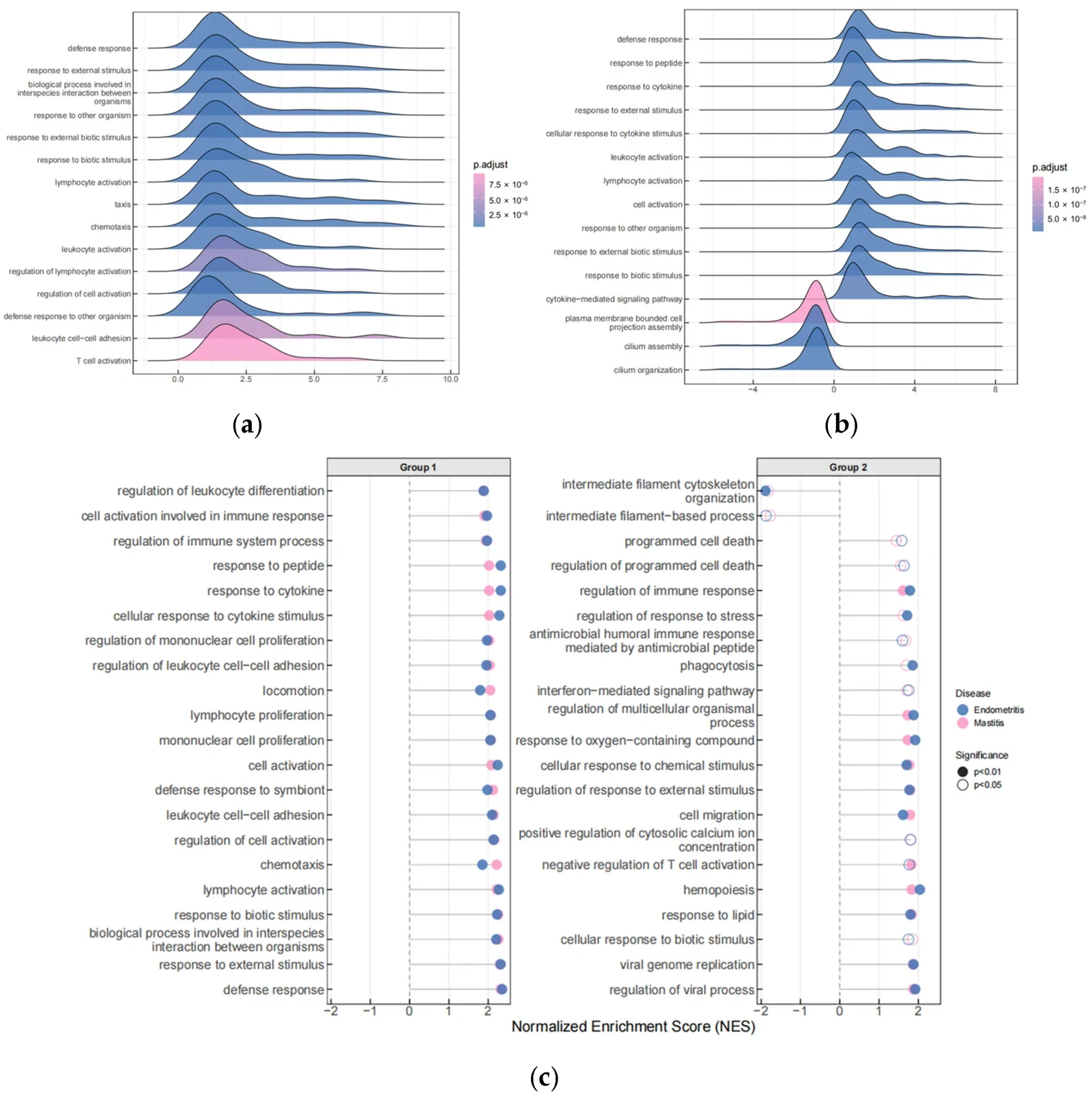
GSEA enrichment profiles and shared pathway intersection of mastitis and endometritis. (**a**) GSEA enrichment results of the mastitis dataset (GSE75379). (**b**) GSEA enrichment results of the endometritis dataset (GSE125964). (**c**) Intersection of the GSEA pathways shared by the two diseases after semantic redundancy reduction.

**Figure 3:**
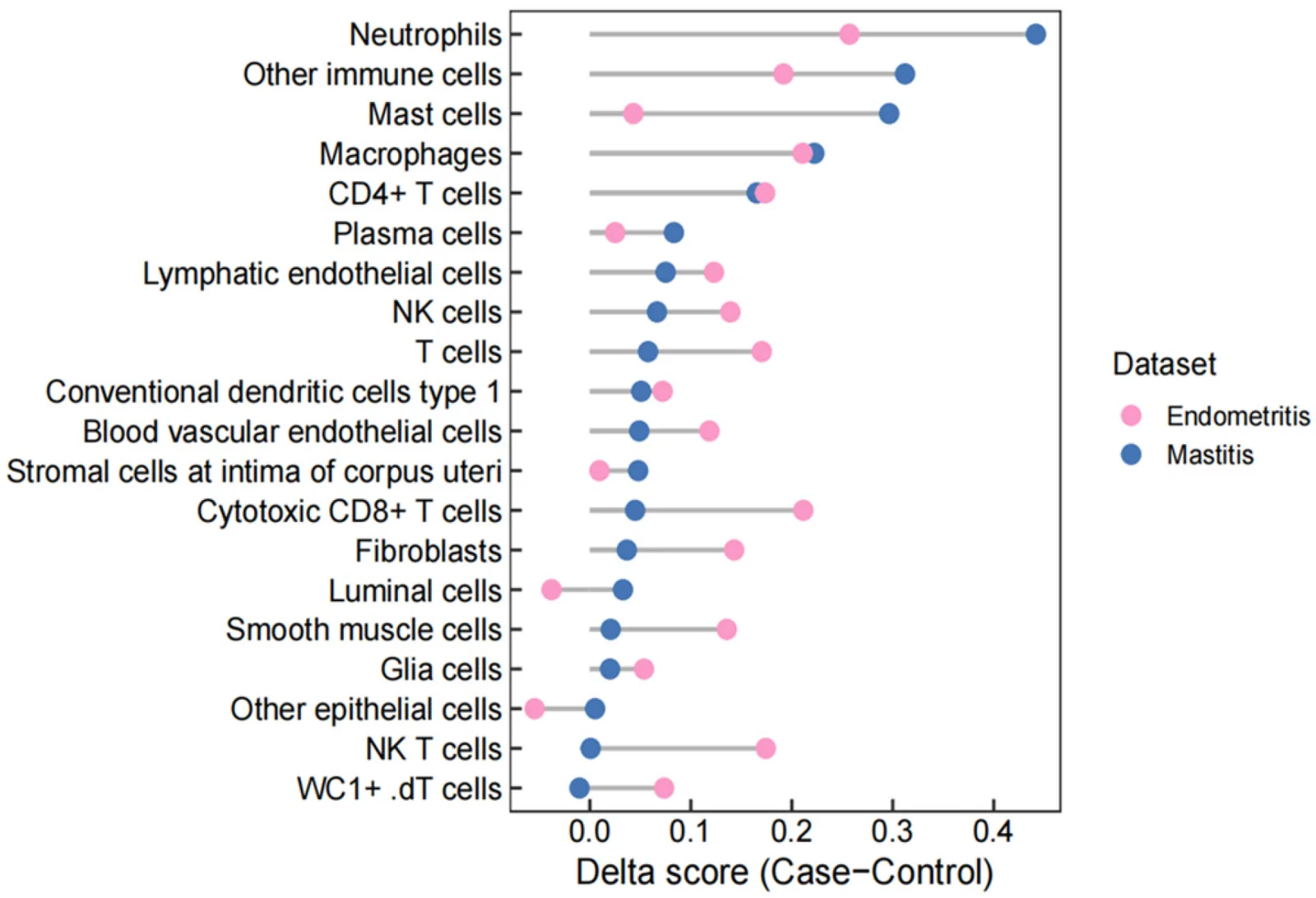
Changes in ssGSEA-based cell-type signature enrichment scores (delta scores) in the two diseases.

**Figure 4:**
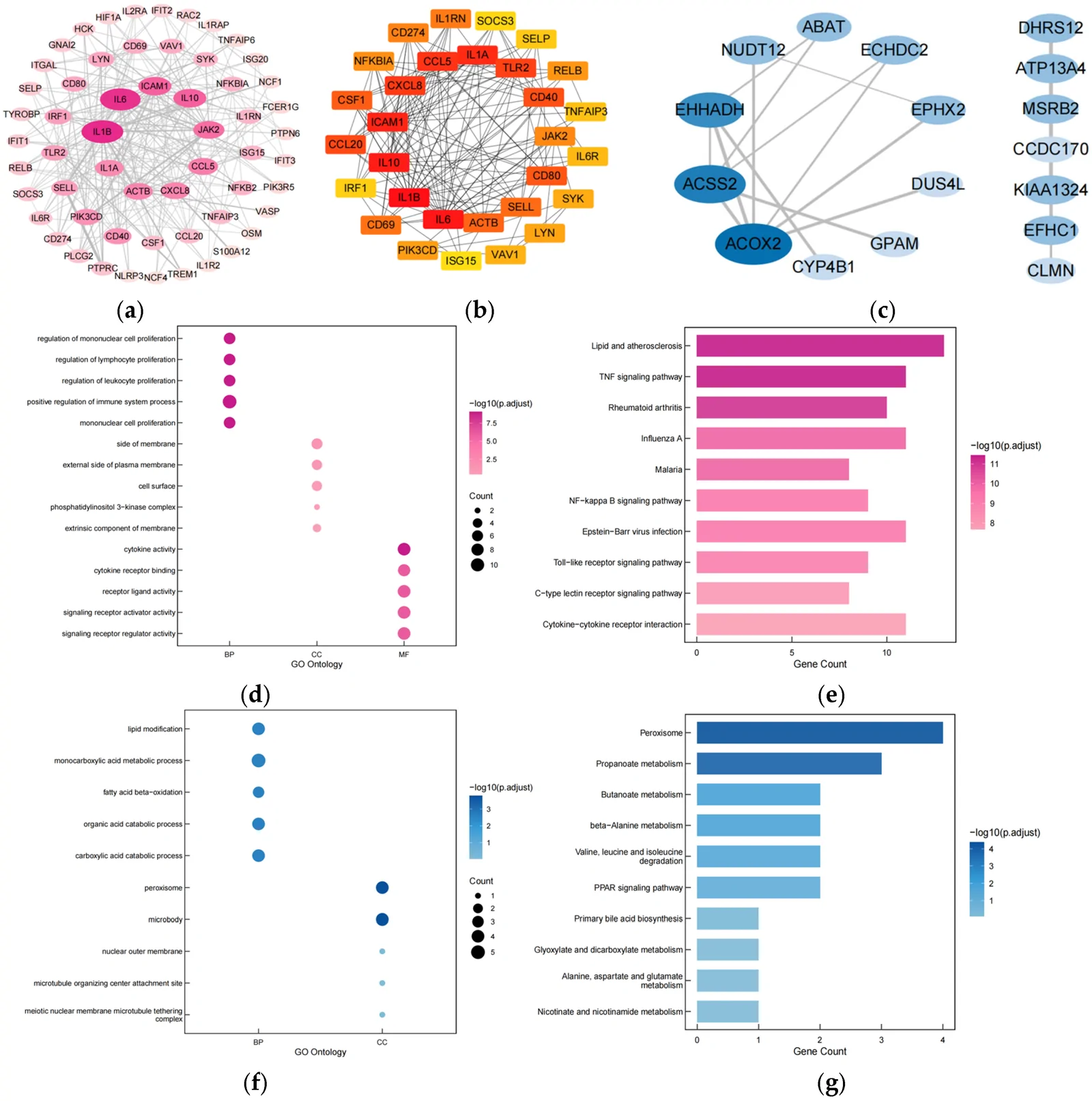
PPI networks and functional enrichment analysis of core shared genes. (**a**) PPI network of the co-upregulated genes (degree > 3). (**b**) Top 30 hub genes screened by the MCC algorithm. (**c**) PPI network of the co-downregulated genes. (**d**) GO enrichment of upregulated core genes. (**e**) KEGG enrichment of upregulated core genes. (**f**) GO enrichment of downregulated core genes. (**g**) KEGG enrichment of downregulated core genes.

**Figure 5:**
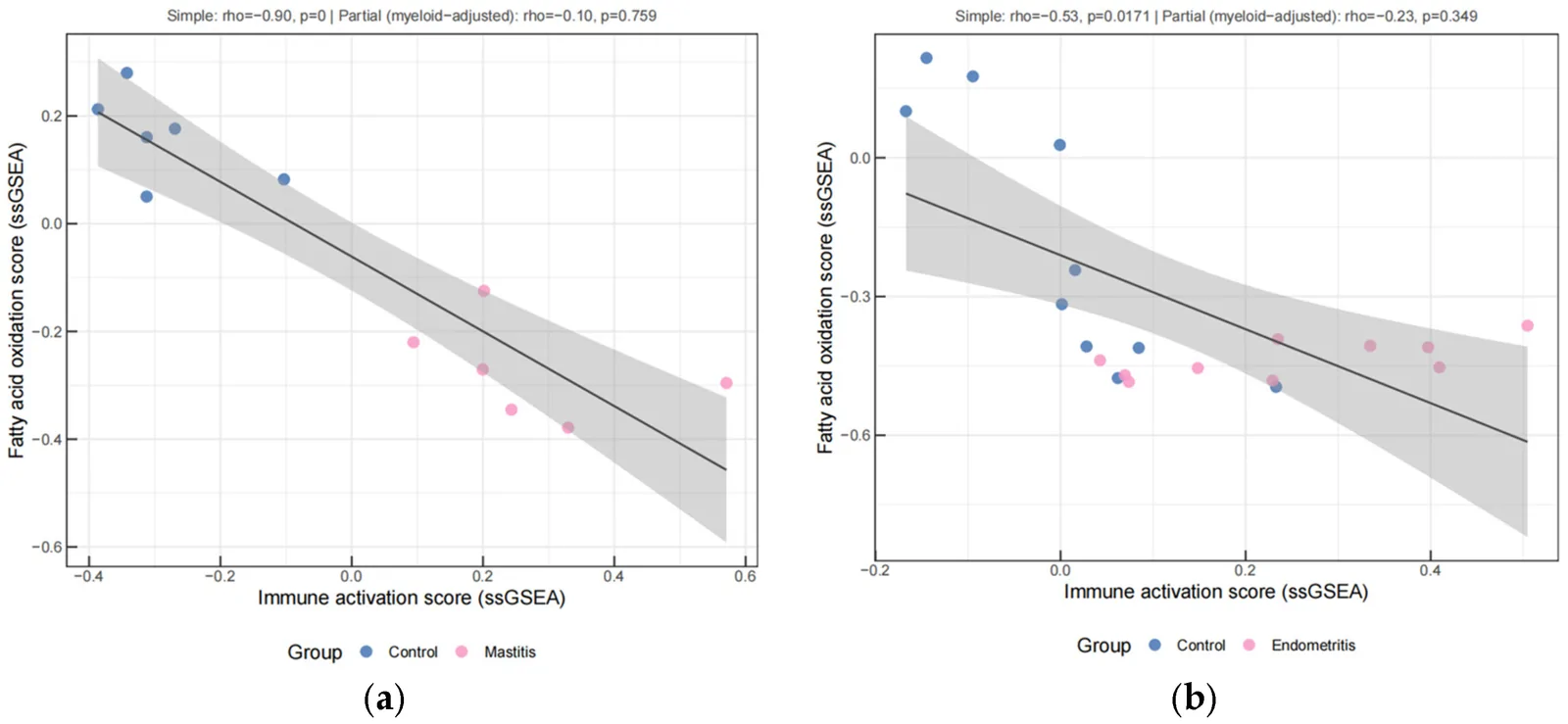
Immune activation–fatty acid oxidation coupling analysis. (**a**) Mastitis dataset. (**b**) Endometritis dataset.

**Figure 6:**
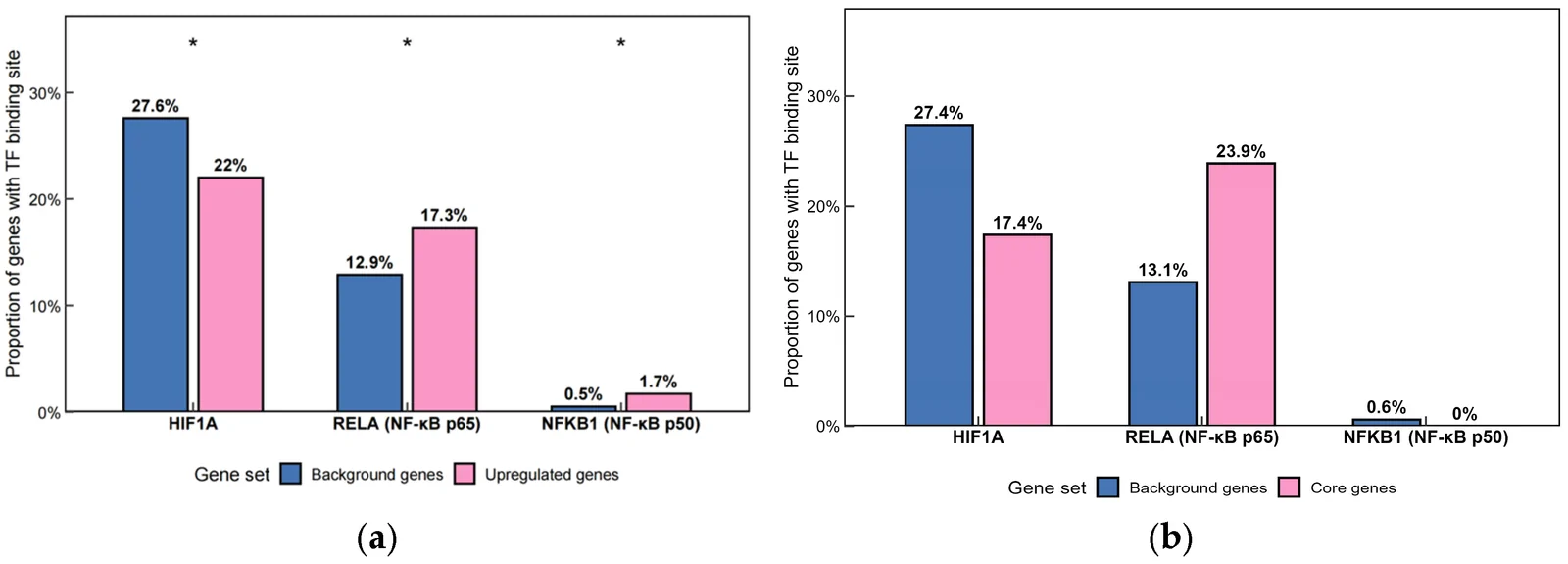
Promoter binding motif enrichment of key transcription factors across two gene sets. (**a**) Binding motif enrichment of *HIF1A*, *RELA* (NF-κB p65), and *NFKB1* (NF-κB p50) in promoters of the 383 concordantly upregulated genes. (**b**) Binding motif enrichment of *HIF1A*, *RELA* (NF-κB p65), and *NFKB1* (NF-κB p50) in promoters of the 47 core shared genes. Asterisks indicate the significance of enrichment (*: *p* < 0.05).

**Figure 7:**
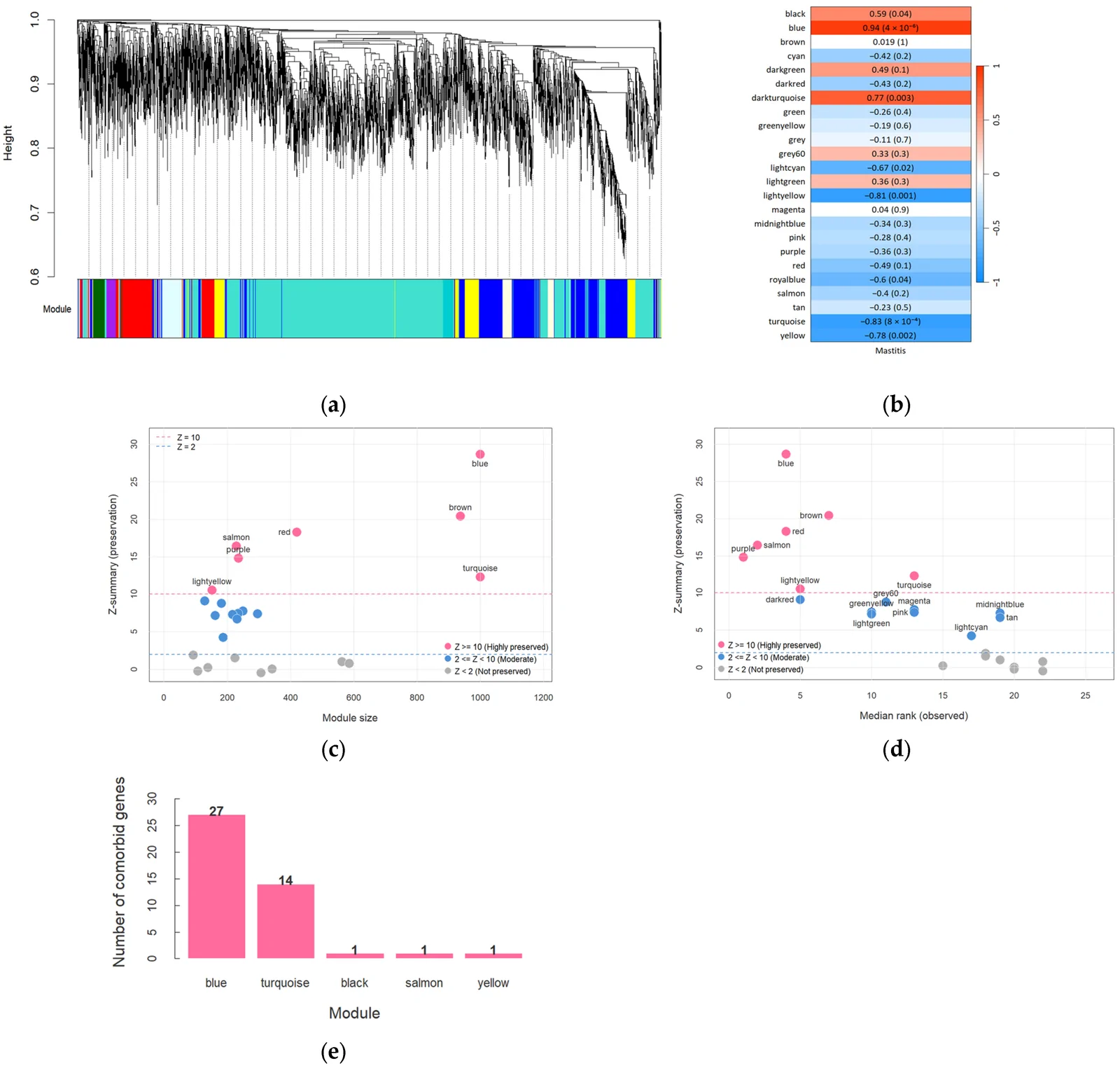
WGCNA co-expression network construction, cross-dataset module preservation, and core shared gene enrichment. (**a**) Gene dendrogram and module color assignment of the mastitis reference network (GSE75379). The colored row beneath the dendrogram indicates the module assignment of each gene, with each color denoting one co-expression module (colors follow the standard WGCNA labeling). (**b**) Module-trait association between co-expression modules and mastitis disease status. (**c**) Cross-dataset module preservation evaluated by Z-summary statistic, with mastitis as the reference network and endometritis as the test network. (**d**) Module preservation statistics assessed by median rank as an independent evaluation metric. (**e**) Distribution of core shared genes across the identified WGCNA modules.

**Figure 8:**
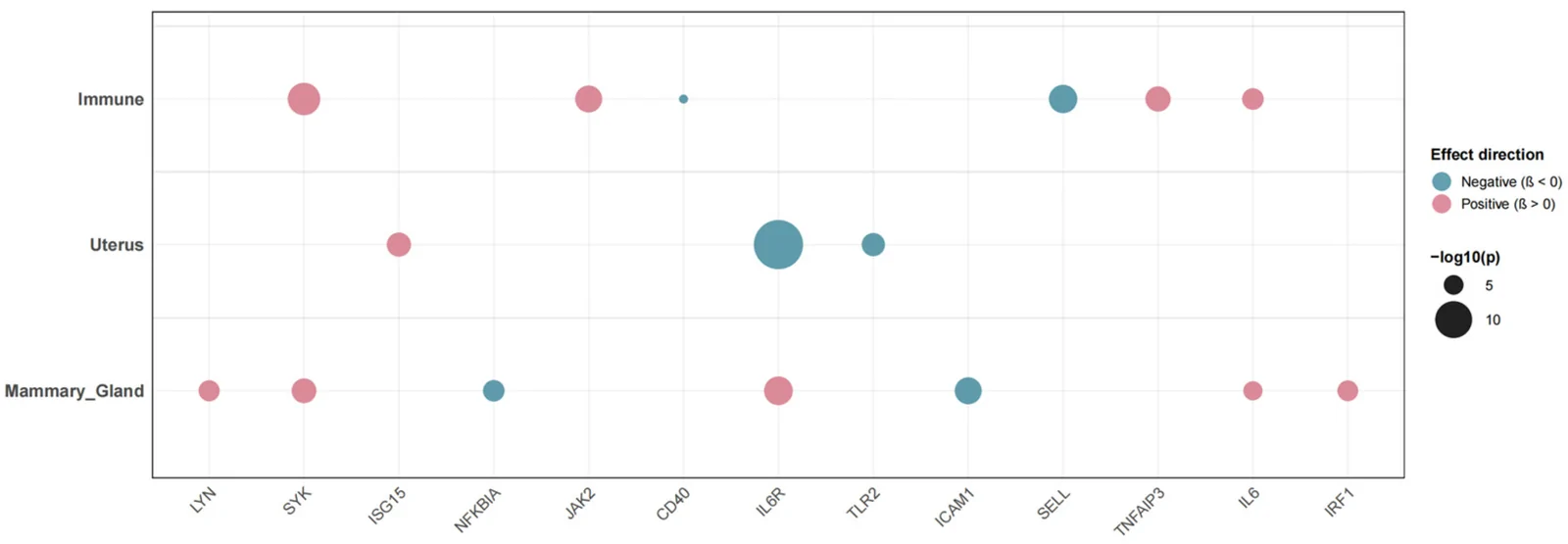
Bubble plot of CattleGTEx eGene validation.

**Figure 9:**
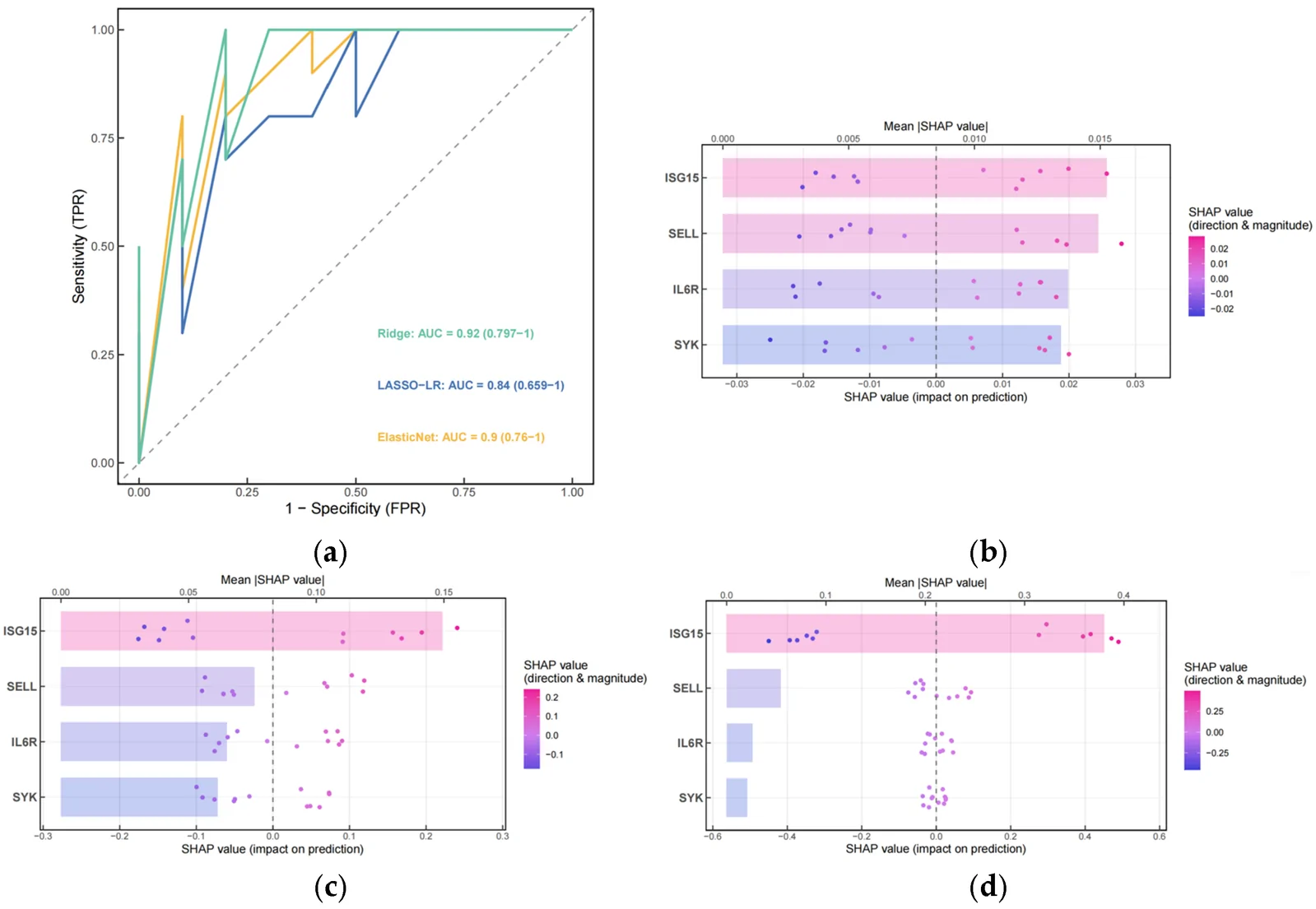
Cross-dataset predictive performance and SHAP interpretability of regularized logistic regression models. (**a**) ROC curves of the Ridge, ElasticNet, and LASSO-LR models in the endometritis transfer cohort (GSE125964). (**b**) SHAP interpretability analysis of the Ridge model. (**c**) SHAP interpretability analysis of the ElasticNet model. (**d**) SHAP interpretability analysis of the LASSO-LR model.

**Figure 10:**
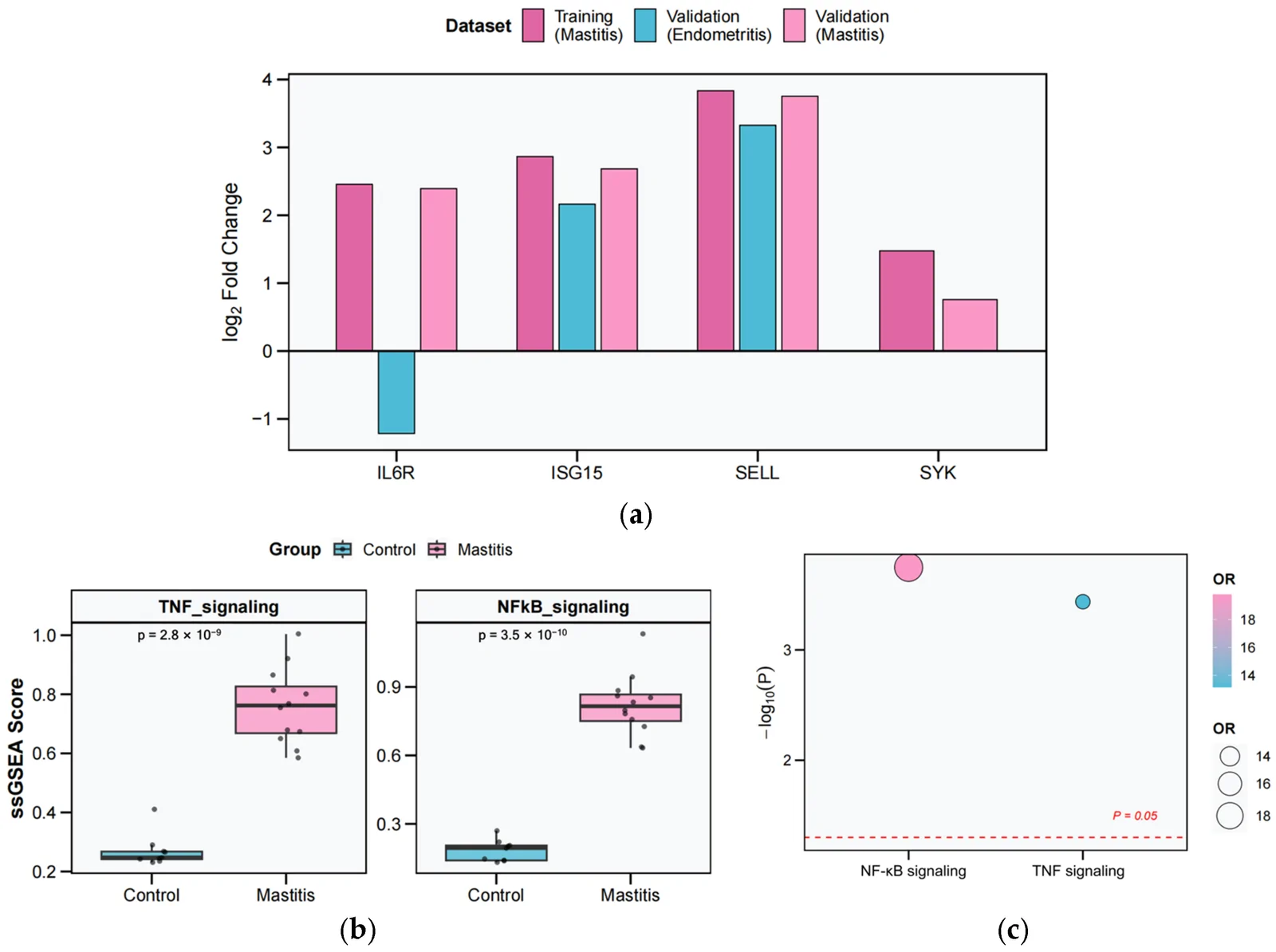
External cross-cohort validation of core genes and inflammatory pathway signatures. (**a**) Log_2_ fold changes of the four raw consensus candidates across the discovery mastitis cohort and the two independent validation cohorts (mastitis GSE24217 and the endometrial summary dataset). (**b**) ssGSEA-derived activation scores of the TNF and NF-κB signaling pathways in the independent mastitis dataset. (**c**) Over-representation analysis of the TNF and NF-κB signaling pathways in the independent endometritis dataset.

**Figure 11:**
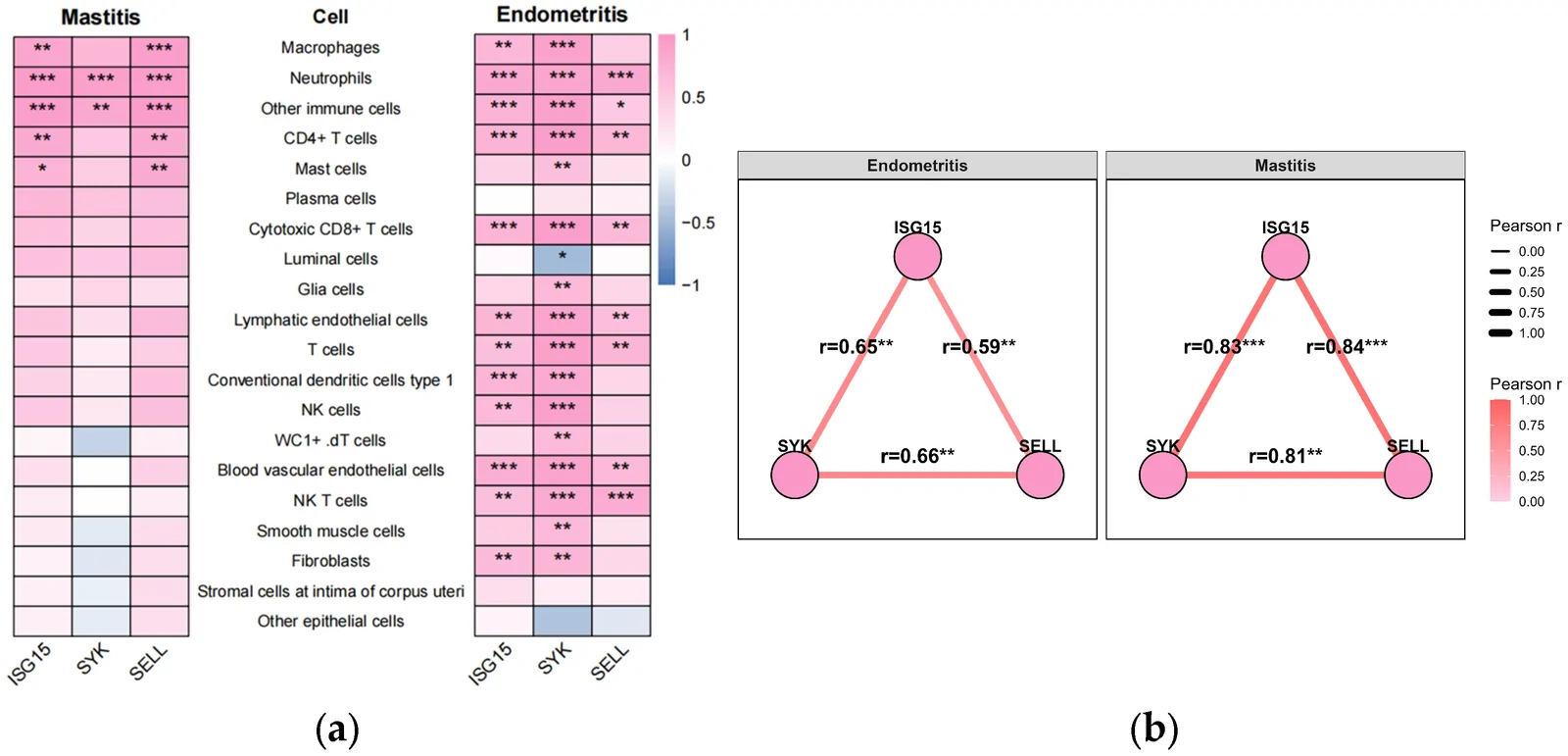
Immune cell correlation profiles and co-expression network of the three core genes. (*: *p* < 0.05, **: *p* < 0.01, ***: *p* < 0.001). (**a**) Pearson correlation heatmaps of core genes and immune cell ssGSEA scores in the mastitis and endometritis datasets. (**b**) Co-expression network of the three core genes.

**Table 1:** In silico candidate modulatory agents for the three core shared genes.

Core Gene	Candidate Traditional Chinese Medicines	Candidate Approved Western Drugs
*SYK*	*Dictamni Cortex*; processed *Aconiti Radix* (*Aconiti Radix Cocta*)	FOSTAMATINIB DISODIUM; FOSTAMATINIB
*ISG15*	Yu–Ping–Feng San	IRINOTECAN HYDROCHLORIDE (no inflammatory indication)
*SELL*	*Ononis spinosa*	No approved targeted drug

## Data Availability

Publicly available datasets were analyzed in this study. Bovine mastitis and endometritis transcriptomic datasets were retrieved from the Gene Expression Omnibus (GEO) database (https://www.ncbi.nlm.nih.gov/geo/, accessed on 1 July 2026) under accession numbers GSE75379, GSE125964 and GSE24217. CattleGTEx cis-eQTL data were downloaded from the official portal (https://cattlegtex.kiz.ac.cn/, accessed on 8 July 2026). Drug–gene interaction data were obtained from DGIdb 5.0 (https://beta.dgidb.org/, accessed on 25 July 2026) and HERB 2.0 (http://47.92.70.12/, accessed on 25 July 2026).

## Supplementary Materials

The following supporting information can be downloaded at https://www.mdpi.com/article/10.3390/cimb48101005/s1.
