# Supplementary material for: Integrated Transcriptomic Analysis Reveals Conserved Inflammatory Transcriptional Signatures Across Bovine Mastitis and Endometritis: Shared Pathways and In Silico Candidate Agents
Source: Curr Issues Mol Biol. 2026 Sep 30;48(10):1005. doi: 10.3390/cimb48101005 (PMC13703076; doi:10.3390/cimb48101005)
Supplement: Supplementary file 1 [file cimb-48-01005-s001.zip › cimb-4548417-supplementary.pdf]

Supplementary Materials

Manuscript ID: CIMB-4548417 | Current Issues in Molecular Biology

List of Supplementary Tables

Table S1. Dataset summary of the discovery and independent validation cohorts.

Table S2. Complete list of the 444 directionally concordant shared DEGs (383 co-upregulated and 61 co-downregulated).

Table S3. Stepwise WGCNA, cis-eQTL, and machine-learning annotation of the 47 core comorbidity genes.

Table S4. The 27 shared comorbidity genes contained in the preserved blue WGCNA module.

Table S5. Per-signature ssGSEA statistics and detected-marker counts in the two discovery datasets.

Table S6a. Three-algorithm consensus selection frequency across 1,000 stratified bootstrap resamples.

Table S6b. Per-model selection count and coefficient-direction stability across 1,000 bootstrap resamples.

Table S6c. Cross-disease transfer AUC (mastitis-trained models applied to the endometritis cohort) with 95% confidence intervals.

Table S7a. Per-gene log2 fold change, P value, and directional concordance in the two independent validation cohorts.

Table S7b. Direction-aware Stouffer Z statistics and module AUC for the four raw candidates and the final three-gene module.

Table S8a. Complete DGIdb 5.0 drug–gene records with approval status, interaction score, and filtering flag.

Table S8b. Retained HERB 2.0 literature-supported traditional Chinese medicine candidates.

Table S1. Dataset summary of the discovery and independent validation cohorts.

Dataset	Tissue/ cell type	Disease definition	Pathogen/ challenge	Postpartum stage	n_control	n_case	Platform	Breed	Parity	DEG/processing used in this study
GSE75379	Mammary/udder tissue	E. coli-induced mastitis (24 h)	E. coli challenge	Early lactation (~4-6 weeks postpartum)	6	6	Illumina HiSeq 2000, 100 bp paired-end (GPL15749)	Not reported in GEO record	Not reported in GEO record	Raw counts; DESeq2 (~CowID+group); padj<0.05 & |log2FC|>1 -> 1924 DEGs (1231 up, 693 down)
GSE125964	Endometrial epithelial cells	Clinical endometritis	Naturally occurring / field	7 days postpartum	10	10	Illumina HiSeq 2500, TruSeq Stranded mRNA (GPL19172)	Holstein-Friesian	Mixed parity	Source-study DEG table used directly (not re-run by DESeq2); FDR<0.05 & |logFC|>1 -> 1296 DEGs (748 up, 548 down)
GSE24217	Mammary gland tissue	E. coli mastitis (24 h)	E. coli challenge	4-6 weeks postpartum	9	12	Affymetrix Bovine Genome Array microarray (GPL2112)	Danish Holstein-Friesian	Primiparous	Gene-level direction, Stouffer Z, module-score AUC, ssGSEA pathway scores
Xie et al. [ref 25]	Superficial endometrial cells	Clinical endometritis	Naturally occurring / field	35-36 days postpartum (5th week)	12	6	Illumina NextSeq 500, 75 bp single-end	Holstein	Multiparous	Source summary DEG table (healthy vs CE; DESeq2, P<0.05 & |FC|>2); direction check + pathway ORA; no individual-level classifier refit

Table S2. Complete list of the 444 directionally concordant shared DEGs (383 co-upregulated and 61 co-downregulated).

Gene	Ensembl_ID	Class	Mastitis_log2FC	Mastitis_padj	Endometritis_log2FC	Endometritis_FDR
SLC11A1	ENSBTAG00000015520	Concordantly up	5.543	7.69e-72	2.076	2.91e-02
ADGRE1	ENSBTAG00000007901	Concordantly up	7.757	2.60e-64	5.339	4.27e-04
	ENSBTAG00000019428	Concordantly up	6.125	1.25e-54	2.481	1.45e-02
	ENSBTAG00000004043	Concordantly up	5.790	1.31e-53	2.936	7.76e-03
SRGN	ENSBTAG00000017863	Concordantly up	5.303	6.26e-53	4.411	2.27e-04
	ENSBTAG00000006354	Concordantly up	5.463	1.77e-47	4.449	4.48e-04
ADGRE3	ENSBTAG00000002937	Concordantly up	7.201	1.56e-42	3.367	1.23e-03
PTAFR	ENSBTAG00000027051	Concordantly up	4.618	2.21e-41	2.776	1.03e-03
	ENSBTAG00000025250	Concordantly up	5.998	1.49e-40	3.943	8.10e-03
IL1RAP	ENSBTAG00000013205	Concordantly up	3.857	1.77e-39	3.144	1.91e-03
	ENSBTAG00000018165	Concordantly up	4.413	1.65e-38	4.346	1.79e-04
	ENSBTAG00000045880	Concordantly up	4.658	2.02e-37	4.385	1.44e-04
HCK	ENSBTAG00000007932	Concordantly up	4.836	1.10e-36	3.295	3.32e-04
PTX3	ENSBTAG00000009012	Concordantly up	4.843	7.21e-36	7.140	1.06e-04
NAIP	ENSBTAG00000003326	Concordantly up	4.837	2.35e-34	3.808	1.75e-04
UPP1	ENSBTAG00000008428	Concordantly up	5.066	1.04e-33	3.580	4.39e-03
SELPLG	ENSBTAG00000031851	Concordantly up	3.269	4.63e-32	1.667	1.07e-02
NFAM1	ENSBTAG00000005018	Concordantly up	4.516	3.21e-31	3.629	9.24e-04
	ENSBTAG00000004556	Concordantly up	3.458	5.19e-31	2.668	2.43e-02
BATF3	ENSBTAG00000011982	Concordantly up	5.049	1.59e-30	4.151	1.90e-04
	ENSBTAG00000019708	Concordantly up	5.386	1.55e-29	2.267	8.63e-03
FYB	ENSBTAG00000001492	Concordantly up	3.612	3.89e-29	3.295	5.29e-04
DUSP2	ENSBTAG00000020350	Concordantly up	4.930	7.43e-29	3.554	1.73e-03
C5AR1	ENSBTAG00000020872	Concordantly up	3.773	2.33e-28	2.588	1.70e-03
TARM1	ENSBTAG00000045861	Concordantly up	5.936	1.30e-27	5.977	1.60e-04
ARG2	ENSBTAG00000001945	Concordantly up	2.323	1.30e-27	3.648	7.98e-04
IFIT3	ENSBTAG00000009768	Concordantly up	3.937	1.76e-27	3.556	3.78e-03

Gene	Ensembl_ID	Class	Mastitis_log2FC	Mastitis_padj	Endometritis_log2FC	Endometritis_FDR
RUBCNL	ENSBTAG00000013371	Concordantly up	4.996	2.94e-27	5.899	1.09e-04
VAT1L	ENSBTAG00000018146	Concordantly up	4.852	5.93e-27	3.423	4.46e-03
DGAT2	ENSBTAG00000001154	Concordantly up	4.382	1.06e-26	5.815	1.06e-04
TMEM156	ENSBTAG00000015649	Concordantly up	2.935	2.06e-26	2.983	9.35e-04
FCAR	ENSBTAG00000021647	Concordantly up	5.814	1.06e-25	3.214	9.09e-03
CYTH4	ENSBTAG00000014237	Concordantly up	4.119	2.87e-25	3.075	9.35e-04
ADAM8	ENSBTAG00000023832	Concordantly up	4.203	3.14e-25	3.420	4.94e-03
FCER1G	ENSBTAG00000024503	Concordantly up	3.119	1.05e-24	2.231	6.76e-03
SELL	ENSBTAG00000011515	Concordantly up	3.834	1.88e-24	4.870	8.36e-05
	ENSBTAG00000006859	Concordantly up	4.023	5.44e-24	4.584	1.75e-04
MEFV	ENSBTAG00000019123	Concordantly up	4.743	5.48e-24	7.739	1.75e-04
	ENSBTAG00000023648	Concordantly up	7.726	1.57e-22	5.465	3.42e-04
FMNL1	ENSBTAG00000004196	Concordantly up	3.125	1.85e-22	2.545	3.98e-03
PLEK	ENSBTAG00000009658	Concordantly up	4.550	5.21e-22	6.463	2.11e-04
S100A8	ENSBTAG00000012640	Concordantly up	7.331	8.11e-22	3.942	1.68e-03
TGM2	ENSBTAG00000016208	Concordantly up	2.366	3.62e-21	2.103	5.55e-03
C5AR2	ENSBTAG00000037735	Concordantly up	3.273	5.68e-21	1.743	7.64e-03
GPR84	ENSBTAG00000015592	Concordantly up	6.725	1.58e-20	9.543	9.20e-05
CSF3R	ENSBTAG00000013334	Concordantly up	7.937	2.12e-20	5.417	3.01e-04
	ENSBTAG00000038042	Concordantly up	6.145	2.91e-20	4.956	1.06e-03
S100A12	ENSBTAG00000012638	Concordantly up	6.277	4.97e-20	3.346	2.55e-03
	ENSBTAG00000045854	Concordantly up	4.211	5.95e-20	2.379	2.00e-02
DMXL2	ENSBTAG00000000737	Concordantly up	6.641	6.06e-20	2.466	1.60e-03
RASGRP4	ENSBTAG00000006998	Concordantly up	3.956	7.40e-20	3.697	4.78e-04
FAM111A	ENSBTAG00000002450	Concordantly up	4.184	8.06e-20	3.899	1.79e-04
PSTPIP1	ENSBTAG00000021144	Concordantly up	2.711	1.15e-19	2.220	1.24e-02
CCRL2	ENSBTAG00000006155	Concordantly up	4.433	1.22e-19	2.700	1.07e-03
CD48	ENSBTAG00000011238	Concordantly up	3.503	1.38e-19	3.046	4.57e-04
IL2RA	ENSBTAG00000020892	Concordantly up	3.765	1.39e-19	4.606	1.55e-04

Gene	Ensembl_ID	Class	Mastitis_log2FC	Mastitis_padj	Endometritis_log2FC	Endometritis_FDR
SLC16A3	ENSBTAG00000017461	Concordantly up	3.085	3.22e-19	1.742	2.52e-02
AMPD3	ENSBTAG00000015821	Concordantly up	3.697	6.81e-19	3.332	2.85e-04
ISG15	ENSBTAG00000014707	Concordantly up	2.864	8.54e-19	3.455	5.23e-03
TYROBP	ENSBTAG00000007338	Concordantly up	3.048	1.66e-18	1.723	3.27e-02
THEMIS2	ENSBTAG00000006223	Concordantly up	3.941	2.47e-18	3.005	1.99e-03
FGR	ENSBTAG00000011784	Concordantly up	2.112	2.59e-18	3.153	6.93e-04
MXD1	ENSBTAG00000001042	Concordantly up	2.376	3.04e-18	3.770	2.68e-04
IGSF6	ENSBTAG00000018869	Concordantly up	3.524	5.28e-18	3.545	3.39e-04
	ENSBTAG00000000812	Concordantly up	4.111	9.31e-18	2.997	1.30e-03
EVI2B	ENSBTAG00000009353	Concordantly up	2.184	1.05e-17	2.329	1.06e-03
CLEC12A	ENSBTAG00000012815	Concordantly up	3.217	1.07e-17	2.353	1.86e-03
GSAP	ENSBTAG00000018430	Concordantly up	1.781	1.07e-17	1.521	1.08e-02
SYK	ENSBTAG00000004767	Concordantly up	1.475	1.18e-17	1.648	6.38e-03
P2RY13	ENSBTAG00000031397	Concordantly up	4.281	1.52e-17	2.972	4.74e-03
	ENSBTAG00000046324	Concordantly up	1.566	1.62e-17	1.635	9.10e-03
SGK1	ENSBTAG00000004269	Concordantly up	2.058	1.86e-17	1.285	2.91e-02
PGS1	ENSBTAG00000000675	Concordantly up	1.577	2.93e-17	1.352	1.56e-03
ALOX5AP	ENSBTAG00000013201	Concordantly up	3.754	5.05e-17	4.909	3.52e-04
SPI1	ENSBTAG00000021709	Concordantly up	3.329	5.77e-17	2.348	6.53e-03
SAMD9	ENSBTAG00000012894	Concordantly up	2.517	6.87e-17	2.372	3.02e-03
GMFG	ENSBTAG00000005390	Concordantly up	2.248	8.34e-17	1.966	4.57e-03
PLEKHO2	ENSBTAG00000008411	Concordantly up	1.946	9.11e-17	1.435	2.91e-02
	ENSBTAG00000019018	Concordantly up	1.801	1.16e-16	1.455	3.80e-02
SLA	ENSBTAG00000007828	Concordantly up	2.780	2.24e-16	3.356	5.57e-04
	ENSBTAG00000019227	Concordantly up	4.339	4.34e-16	3.655	5.42e-04
IL10	ENSBTAG00000006685	Concordantly up	4.936	4.35e-16	4.385	5.56e-04
SLC39A14	ENSBTAG00000019225	Concordantly up	1.594	5.47e-16	1.642	3.08e-03
S100A9	ENSBTAG00000006505	Concordantly up	7.257	7.61e-16	5.337	1.75e-04
CCL16	ENSBTAG00000007193	Concordantly up	2.892	8.26e-16	3.737	7.14e-04

Gene	Ensembl_ID	Class	Mastitis_log2FC	Mastitis_padj	Endometritis_log2FC	Endometritis_FDR
MKNK1	ENSBTAG00000019933	Concordantly up	1.694	9.19e-16	1.763	1.63e-03
C16orf54	ENSBTAG00000047448	Concordantly up	2.612	2.07e-15	2.262	3.31e-03
PSTPIP2	ENSBTAG00000002624	Concordantly up	2.215	3.36e-15	3.899	1.79e-04
CD53	ENSBTAG00000006466	Concordantly up	2.887	3.72e-15	2.266	3.87e-03
	ENSBTAG00000048184	Concordantly up	4.239	4.60e-15	2.584	1.51e-02
NCF1	ENSBTAG00000003305	Concordantly up	5.196	7.29e-15	3.320	7.15e-04
	ENSBTAG00000001066	Concordantly up	2.942	8.92e-15	2.059	9.10e-03
IL1RN	ENSBTAG00000019665	Concordantly up	5.335	1.04e-14	3.492	5.78e-03
STX11	ENSBTAG00000011236	Concordantly up	3.075	1.47e-14	3.240	3.94e-03
FTH1	ENSBTAG00000011184	Concordantly up	2.302	1.71e-14	1.248	1.57e-02
CD55	ENSBTAG00000006984	Concordantly up	5.337	2.57e-14	2.051	1.15e-02
C12orf75	ENSBTAG00000045742	Concordantly up	3.186	2.70e-14	2.604	3.43e-04
ARHGAP45	ENSBTAG00000020772	Concordantly up	1.835	3.00e-14	1.999	7.72e-03
WDFY4	ENSBTAG00000023028	Concordantly up	2.747	3.67e-14	1.583	3.60e-02
FAM49B	ENSBTAG00000020801	Concordantly up	1.784	3.90e-14	1.577	4.96e-03
	ENSBTAG00000047570	Concordantly up	4.029	4.11e-14	2.628	1.20e-02
IFITM1	ENSBTAG00000019017	Concordantly up	2.267	4.11e-14	1.567	3.41e-02
TGM3	ENSBTAG00000001785	Concordantly up	8.827	4.62e-14	6.374	4.48e-04
	ENSBTAG00000026753	Concordantly up	8.096	5.03e-14	4.920	9.35e-04
PKM	ENSBTAG00000001601	Concordantly up	1.703	5.45e-14	1.203	8.57e-03
	ENSBTAG00000036224	Concordantly up	3.397	6.26e-14	3.805	5.21e-04
FXYD5	ENSBTAG00000031441	Concordantly up	2.248	6.31e-14	1.710	1.14e-02
RHOG	ENSBTAG00000000347	Concordantly up	1.353	7.91e-14	1.693	9.88e-03
SDC4	ENSBTAG00000015127	Concordantly up	2.433	8.05e-14	1.504	7.79e-03
LCP1	ENSBTAG00000007079	Concordantly up	2.527	8.76e-14	2.134	3.85e-03
TREM1	ENSBTAG00000017593	Concordantly up	7.251	1.74e-13	3.912	3.86e-03
RETN	ENSBTAG00000004716	Concordantly up	6.085	2.13e-13	4.737	6.09e-04
BIRC3	ENSBTAG00000024918	Concordantly up	2.564	2.51e-13	3.467	4.66e-04
PDE4B	ENSBTAG00000008636	Concordantly up	2.102	2.53e-13	4.568	1.63e-04

Gene	Ensembl_ID	Class	Mastitis_log2FC	Mastitis_padj	Endometritis_log2FC	Endometritis_FDR
	ENSBTAG00000001143	Concordantly up	2.108	2.57e-13	3.693	3.52e-04
CCDC88B	ENSBTAG00000040190	Concordantly up	2.592	2.73e-13	2.029	1.46e-02
RILPL2	ENSBTAG00000007804	Concordantly up	2.223	3.41e-13	1.747	2.69e-03
CSF2RA	ENSBTAG00000045948	Concordantly up	2.202	3.42e-13	1.590	1.73e-02
SLAMF6	ENSBTAG00000014368	Concordantly up	2.545	4.09e-13	3.151	8.27e-04
IL1R2	ENSBTAG00000006343	Concordantly up	6.055	4.20e-13	6.121	3.41e-04
ARHGAP30	ENSBTAG00000017875	Concordantly up	2.109	4.53e-13	1.789	5.70e-03
CHST15	ENSBTAG00000003312	Concordantly up	2.295	4.89e-13	1.714	1.96e-02
RGS19	ENSBTAG00000021837	Concordantly up	1.690	1.03e-12	1.671	2.58e-03
TBC1D9	ENSBTAG00000018951	Concordantly up	1.466	1.12e-12	1.149	2.73e-02
	ENSBTAG00000037800	Concordantly up	6.275	1.42e-12	5.921	2.78e-04
IL6R	ENSBTAG00000018474	Concordantly up	2.456	1.49e-12	1.009	1.56e-02
IL18RAP	ENSBTAG00000033748	Concordantly up	4.563	1.54e-12	5.604	1.06e-04
	ENSBTAG00000048155	Concordantly up	6.492	1.88e-12	4.495	2.60e-04
ISG20	ENSBTAG00000014762	Concordantly up	1.767	1.95e-12	3.760	9.35e-04
ACOD1	ENSBTAG00000008259	Concordantly up	5.797	1.96e-12	3.160	5.76e-03
	ENSBTAG00000014953	Concordantly up	3.803	2.48e-12	4.224	6.09e-04
MCEMP1	ENSBTAG00000004714	Concordantly up	8.170	2.98e-12	6.702	8.36e-05
MAP3K8	ENSBTAG00000011600	Concordantly up	2.015	3.25e-12	2.164	4.39e-03
GMIP	ENSBTAG00000015144	Concordantly up	1.829	3.45e-12	1.890	1.76e-03
PTPRE	ENSBTAG00000002435	Concordantly up	2.174	5.16e-12	1.443	1.77e-02
SEMA4D	ENSBTAG00000001941	Concordantly up	1.464	5.76e-12	2.455	1.03e-03
IER5	ENSBTAG00000039764	Concordantly up	2.313	7.28e-12	1.617	2.36e-02
F5	ENSBTAG00000017722	Concordantly up	2.636	7.36e-12	2.113	4.50e-03
NFKBIA	ENSBTAG00000016683	Concordantly up	1.813	7.86e-12	4.176	2.06e-04
TNFRSF1B	ENSBTAG00000024928	Concordantly up	1.876	8.78e-12	1.957	2.26e-03
SSH2	ENSBTAG00000011011	Concordantly up	2.413	8.87e-12	2.156	8.19e-04
MYO1F	ENSBTAG00000007661	Concordantly up	1.808	9.03e-12	1.911	4.32e-03
CREM	ENSBTAG00000016060	Concordantly up	1.954	1.20e-11	2.776	1.47e-03

Gene	Ensembl_ID	Class	Mastitis_log2FC	Mastitis_padj	Endometritis_log2FC	Endometritis_FDR
CD80	ENSBTAG00000018059	Concordantly up	2.971	1.24e-11	2.205	7.63e-03
APBB1IP	ENSBTAG00000012526	Concordantly up	2.265	1.35e-11	1.324	4.43e-02
ETS2	ENSBTAG00000009214	Concordantly up	1.629	1.60e-11	1.660	2.86e-03
	ENSBTAG00000008851	Concordantly up	7.429	2.13e-11	6.544	2.11e-04
ECM1	ENSBTAG00000003806	Concordantly up	2.515	2.43e-11	2.005	1.31e-02
TIAM2	ENSBTAG00000010047	Concordantly up	2.477	2.52e-11	4.990	1.53e-04
ZDHHC2	ENSBTAG00000044194	Concordantly up	3.035	2.67e-11	3.300	4.48e-04
	ENSBTAG00000011044	Concordantly up	2.887	2.68e-11	3.371	3.30e-04
CCL5	ENSBTAG00000007191	Concordantly up	2.533	2.93e-11	3.090	4.59e-03
PLAU	ENSBTAG00000005947	Concordantly up	2.700	3.59e-11	3.389	1.10e-03
LIMD2	ENSBTAG00000008154	Concordantly up	1.423	4.25e-11	1.423	1.37e-02
ICAM3	ENSBTAG00000015742	Concordantly up	1.235	5.22e-11	4.293	2.33e-04
RAC2	ENSBTAG00000011043	Concordantly up	2.409	5.59e-11	2.427	9.35e-04
VMP1	ENSBTAG00000011623	Concordantly up	1.982	5.97e-11	1.304	1.81e-02
PREX1	ENSBTAG00000015609	Concordantly up	2.536	5.99e-11	2.848	4.48e-04
BST1	ENSBTAG00000006156	Concordantly up	6.461	6.18e-11	5.084	1.12e-04
COTL1	ENSBTAG00000016315	Concordantly up	1.809	6.40e-11	1.891	1.94e-03
ITGAL	ENSBTAG00000007103	Concordantly up	2.207	7.20e-11	1.468	1.89e-02
PLAC8	ENSBTAG00000031750	Concordantly up	2.031	8.47e-11	1.278	4.09e-02
CYP27B1	ENSBTAG00000016906	Concordantly up	3.227	8.49e-11	3.076	3.78e-03
VAV1	ENSBTAG00000039160	Concordantly up	2.886	1.11e-10	1.455	5.38e-03
RYR1	ENSBTAG00000006999	Concordantly up	2.439	1.24e-10	3.122	1.20e-03
TMEM176B	ENSBTAG00000016766	Concordantly up	1.903	1.47e-10	2.105	1.59e-02
NLRP3	ENSBTAG00000001273	Concordantly up	3.451	1.48e-10	5.467	1.61e-04
AOAH	ENSBTAG00000018119	Concordantly up	2.911	1.49e-10	3.896	9.35e-04
NCF2	ENSBTAG00000008004	Concordantly up	3.425	1.62e-10	2.293	7.19e-03
	ENSBTAG00000015061	Concordantly up	6.553	1.71e-10	3.831	5.35e-03
VASP	ENSBTAG00000019604	Concordantly up	1.211	2.34e-10	1.367	7.06e-03
EHD1	ENSBTAG00000007361	Concordantly up	1.331	2.56e-10	1.180	1.57e-02

Gene	Ensembl_ID	Class	Mastitis_log2FC	Mastitis_padj	Endometritis_log2FC	Endometritis_FDR
	ENSBTAG00000027760	Concordantly up	2.226	2.72e-10	1.548	1.50e-02
JAK2	ENSBTAG00000012047	Concordantly up	1.341	3.03e-10	1.561	5.60e-03
CPNE2	ENSBTAG00000002233	Concordantly up	2.347	3.41e-10	1.739	3.52e-03
SCNN1D	ENSBTAG00000017674	Concordantly up	2.904	3.74e-10	2.397	5.96e-03
FGD3	ENSBTAG00000006939	Concordantly up	1.911	5.22e-10	2.313	8.61e-04
	ENSBTAG00000033565	Concordantly up	2.176	5.41e-10	2.290	1.07e-02
EHBP1L1	ENSBTAG00000009554	Concordantly up	1.229	6.15e-10	1.149	1.06e-02
OSM	ENSBTAG00000016163	Concordantly up	5.069	7.56e-10	6.504	4.78e-04
SOCS3	ENSBTAG00000008441	Concordantly up	4.426	8.03e-10	4.664	4.66e-04
NFIL3	ENSBTAG00000017763	Concordantly up	1.342	1.00e-09	3.311	3.46e-04
ICAM1	ENSBTAG00000010303	Concordantly up	2.183	1.01e-09	1.674	1.00e-02
PTPN6	ENSBTAG00000020294	Concordantly up	1.032	1.52e-09	1.107	4.29e-02
	ENSBTAG00000014731	Concordantly up	1.649	1.71e-09	1.271	4.94e-02
	ENSBTAG00000027513	Concordantly up	5.530	1.72e-09	4.538	1.03e-03
TREML2	ENSBTAG00000015707	Concordantly up	6.296	1.99e-09	3.898	4.50e-03
IL10RA	ENSBTAG00000005215	Concordantly up	2.205	2.02e-09	1.828	7.03e-03
LTB	ENSBTAG00000020674	Concordantly up	2.859	2.22e-09	4.040	2.11e-04
FEM1C	ENSBTAG00000008699	Concordantly up	1.152	2.47e-09	1.497	2.55e-03
PIM1	ENSBTAG00000000396	Concordantly up	1.497	2.84e-09	2.351	1.88e-03
LAT2	ENSBTAG00000045767	Concordantly up	2.123	2.86e-09	2.232	2.45e-03
	ENSBTAG00000002773	Concordantly up	3.341	3.08e-09	1.997	4.25e-02
HCLS1	ENSBTAG00000001009	Concordantly up	1.800	3.25e-09	2.614	7.69e-04
UNC13D	ENSBTAG00000013556	Concordantly up	1.743	3.76e-09	1.904	1.30e-02
SNX20	ENSBTAG00000003961	Concordantly up	2.984	4.15e-09	2.369	7.19e-04
KLHL8	ENSBTAG00000002455	Concordantly up	1.324	4.15e-09	2.187	1.99e-03
CORO1A	ENSBTAG00000008631	Concordantly up	2.037	4.16e-09	2.775	1.08e-03
RAB20	ENSBTAG00000031962	Concordantly up	1.587	4.23e-09	3.159	6.31e-04
TNIP3	ENSBTAG00000047107	Concordantly up	7.149	4.42e-09	2.711	4.19e-02
CDA	ENSBTAG00000006136	Concordantly up	6.005	5.39e-09	2.305	1.41e-02

Gene	Ensembl_ID	Class	Mastitis_log2FC	Mastitis_padj	Endometritis_log2FC	Endometritis_FDR
SLC45A4	ENSBTAG00000008597	Concordantly up	1.519	6.23e-09	1.921	7.02e-03
AMPD2	ENSBTAG00000013018	Concordantly up	1.735	7.93e-09	1.725	1.28e-03
HIF1A	ENSBTAG00000020935	Concordantly up	1.133	9.16e-09	1.157	1.60e-02
SH2D3C	ENSBTAG00000015375	Concordantly up	1.228	1.03e-08	2.990	1.16e-03
	ENSBTAG00000006322	Concordantly up	1.017	1.19e-08	1.384	3.79e-03
TNFSF9	ENSBTAG00000046266	Concordantly up	3.298	1.20e-08	3.355	1.39e-03
IL18BP	ENSBTAG00000027676	Concordantly up	3.534	1.22e-08	3.445	6.00e-03
	ENSBTAG00000047904	Concordantly up	3.866	1.30e-08	5.781	9.25e-04
TNFAIP6	ENSBTAG00000007239	Concordantly up	4.638	1.35e-08	4.016	9.29e-04
MAFG	ENSBTAG00000000040	Concordantly up	1.151	1.44e-08	1.058	2.68e-02
TSPO	ENSBTAG00000018073	Concordantly up	1.870	1.46e-08	2.127	8.71e-03
IL1B	ENSBTAG00000001321	Concordantly up	4.993	1.58e-08	6.369	5.70e-04
LPCAT2	ENSBTAG00000019272	Concordantly up	2.488	1.86e-08	3.015	2.44e-04
SAG	ENSBTAG00000021480	Concordantly up	3.598	1.99e-08	4.964	2.78e-04
SLC16A6	ENSBTAG00000003074	Concordantly up	1.944	2.10e-08	1.827	1.77e-02
NFKB2	ENSBTAG00000006017	Concordantly up	1.459	2.37e-08	2.150	1.85e-03
CD40	ENSBTAG00000020736	Concordantly up	3.096	2.37e-08	1.738	1.48e-02
CDKN2D	ENSBTAG00000010731	Concordantly up	1.503	2.46e-08	3.879	2.84e-04
UCP2	ENSBTAG00000003692	Concordantly up	2.399	2.55e-08	2.025	3.32e-03
STX2	ENSBTAG00000007988	Concordantly up	1.611	2.83e-08	1.125	5.39e-03
SLC25A19	ENSBTAG00000000202	Concordantly up	1.166	3.04e-08	1.612	2.03e-03
CXCL8	ENSBTAG00000019716	Concordantly up	5.385	3.18e-08	5.163	1.17e-03
	ENSBTAG00000037558	Concordantly up	3.768	3.79e-08	6.109	1.79e-04
PXN	ENSBTAG00000018108	Concordantly up	1.016	4.21e-08	1.340	3.08e-02
	ENSBTAG00000039520	Concordantly up	1.856	5.93e-08	1.271	4.28e-02
CAPN3	ENSBTAG00000008868	Concordantly up	2.180	6.54e-08	3.523	1.75e-04
IER3	ENSBTAG00000011358	Concordantly up	1.945	6.80e-08	1.312	3.12e-02
	ENSBTAG00000037778	Concordantly up	1.473	7.50e-08	2.641	2.00e-02
IL1RL1	ENSBTAG00000018571	Concordantly up	5.687	8.31e-08	5.930	4.69e-04

Gene	Ensembl_ID	Class	Mastitis_log2FC	Mastitis_padj	Endometritis_log2FC	Endometritis_FDR
TNIP1	ENSBTAG00000012671	Concordantly up	1.131	8.91e-08	1.906	3.13e-03
JAML	ENSBTAG00000023283	Concordantly up	2.189	9.79e-08	1.603	8.69e-03
GPAT3	ENSBTAG00000017592	Concordantly up	1.893	1.21e-07	3.198	1.03e-03
	ENSBTAG00000023026	Concordantly up	4.728	1.60e-07	8.400	1.06e-04
2003-03-01 00:00:00	ENSBTAG00000006797	Concordantly up	1.709	1.87e-07	2.205	9.35e-04
ARID3A	ENSBTAG00000008607	Concordantly up	1.742	1.89e-07	1.367	6.22e-03
	ENSBTAG00000007974	Concordantly up	1.087	1.95e-07	1.341	8.69e-03
HSH2D	ENSBTAG00000038154	Concordantly up	3.106	1.97e-07	3.369	1.88e-03
CLEC4A	ENSBTAG00000038490	Concordantly up	1.903	2.10e-07	1.560	1.96e-02
IL6	ENSBTAG00000014921	Concordantly up	3.723	2.26e-07	2.802	4.95e-03
CD101	ENSBTAG00000018203	Concordantly up	2.206	2.31e-07	2.805	8.61e-04
FMNL3	ENSBTAG00000016593	Concordantly up	1.757	2.41e-07	1.468	4.03e-02
LYN	ENSBTAG00000020034	Concordantly up	1.130	2.87e-07	1.445	1.13e-02
RASSF2	ENSBTAG00000017346	Concordantly up	2.567	3.20e-07	1.944	3.55e-03
ZDHHC18	ENSBTAG00000046672	Concordantly up	1.476	3.30e-07	1.768	4.26e-03
TLR2	ENSBTAG00000008008	Concordantly up	1.654	3.36e-07	1.911	9.87e-03
ADGRG3	ENSBTAG00000006221	Concordantly up	5.572	3.66e-07	7.382	2.80e-04
GNA15	ENSBTAG00000019988	Concordantly up	2.583	3.77e-07	1.592	4.32e-02
DAGLB	ENSBTAG00000009236	Concordantly up	1.360	3.84e-07	1.910	1.20e-03
JUNB	ENSBTAG00000012046	Concordantly up	1.682	4.15e-07	2.996	1.35e-03
RGS2	ENSBTAG00000034366	Concordantly up	1.817	4.52e-07	2.596	4.59e-03
PTPRC	ENSBTAG00000023144	Concordantly up	1.405	4.54e-07	2.036	9.91e-04
ARRDC4	ENSBTAG00000018252	Concordantly up	1.625	4.68e-07	2.472	2.77e-03
SBNO2	ENSBTAG00000020780	Concordantly up	1.456	5.27e-07	1.687	7.03e-03
NABP1	ENSBTAG00000018653	Concordantly up	1.388	5.63e-07	1.113	1.38e-02
MSC	ENSBTAG00000005404	Concordantly up	3.025	7.01e-07	2.035	4.77e-02
GNAI2	ENSBTAG00000020645	Concordantly up	1.204	7.24e-07	1.084	1.28e-02
	ENSBTAG00000017670	Concordantly up	1.261	7.64e-07	2.397	7.44e-03
NIN	ENSBTAG00000020281	Concordantly up	1.282	8.14e-07	1.796	9.91e-04

Gene	Ensembl_ID	Class	Mastitis_log2FC	Mastitis_padj	Endometritis_log2FC	Endometritis_FDR
NFE2	ENSBTAG00000001562	Concordantly up	4.440	9.50e-07	5.502	2.11e-04
CCDC69	ENSBTAG00000002834	Concordantly up	1.372	1.13e-06	1.234	3.04e-02
PIK3CD	ENSBTAG00000018984	Concordantly up	1.676	1.45e-06	2.809	3.42e-04
VCAN	ENSBTAG00000014906	Concordantly up	2.457	1.52e-06	1.579	2.87e-02
RNF122	ENSBTAG00000016411	Concordantly up	2.056	1.59e-06	1.833	7.44e-03
TAGAP	ENSBTAG00000031701	Concordantly up	1.581	1.74e-06	4.798	2.13e-04
SELP	ENSBTAG00000020755	Concordantly up	1.910	1.79e-06	2.634	5.46e-03
SORL1	ENSBTAG00000014611	Concordantly up	1.817	1.80e-06	1.522	1.50e-02
RNASE6	ENSBTAG00000008792	Concordantly up	1.255	1.87e-06	1.581	1.35e-02
	ENSBTAG00000025257	Concordantly up	4.040	2.15e-06	4.678	2.34e-03
RAB8B	ENSBTAG00000011146	Concordantly up	1.198	2.52e-06	1.711	2.05e-03
RELB	ENSBTAG00000038428	Concordantly up	1.166	2.53e-06	1.315	8.56e-03
	ENSBTAG00000046383	Concordantly up	4.282	2.73e-06	2.115	4.74e-02
RIDA	ENSBTAG00000012595	Concordantly up	1.038	2.89e-06	2.100	5.72e-04
PROK2	ENSBTAG00000019330	Concordantly up	5.751	3.45e-06	8.299	5.67e-04
TMEM176A	ENSBTAG00000012078	Concordantly up	1.778	3.68e-06	2.325	8.59e-03
LSP1	ENSBTAG00000010447	Concordantly up	1.067	3.76e-06	2.063	9.10e-03
SUSD3	ENSBTAG00000000181	Concordantly up	1.876	3.90e-06	1.796	5.06e-03
ECSCR	ENSBTAG00000030518	Concordantly up	1.231	4.06e-06	1.037	4.70e-02
	ENSBTAG00000045751	Concordantly up	1.327	4.15e-06	1.429	4.93e-02
ACVRL1	ENSBTAG00000013843	Concordantly up	1.027	4.81e-06	1.330	4.63e-02
TRIB1	ENSBTAG00000023179	Concordantly up	1.283	6.08e-06	2.044	1.17e-03
SPTSSA	ENSBTAG00000013981	Concordantly up	1.041	6.59e-06	1.056	1.53e-02
C10orf54	ENSBTAG00000010888	Concordantly up	1.644	6.82e-06	3.180	4.76e-04
ABCA7	ENSBTAG00000020766	Concordantly up	1.787	7.12e-06	3.331	1.79e-04
SDS	ENSBTAG00000031814	Concordantly up	3.449	7.56e-06	4.826	5.87e-04
IFIT1	ENSBTAG00000007881	Concordantly up	1.920	7.67e-06	2.335	2.48e-02
ICAM2	ENSBTAG00000019432	Concordantly up	1.366	7.98e-06	2.932	8.17e-04
	ENSBTAG00000007213	Concordantly up	1.289	1.01e-05	1.013	3.37e-02

Gene	Ensembl_ID	Class	Mastitis_log2FC	Mastitis_padj	Endometritis_log2FC	Endometritis_FDR
HMGB2	ENSBTAG00000015101	Concordantly up	1.029	1.03e-05	1.724	4.66e-04
GAPT	ENSBTAG00000016770	Concordantly up	2.377	1.26e-05	4.144	1.88e-04
	ENSBTAG00000038067	Concordantly up	4.243	1.35e-05	2.250	3.73e-02
	ENSBTAG00000006523	Concordantly up	3.531	1.41e-05	3.502	1.03e-03
CDKN1A	ENSBTAG00000008353	Concordantly up	1.665	1.48e-05	1.969	3.41e-03
PTPN1	ENSBTAG00000006616	Concordantly up	1.144	1.52e-05	1.606	6.38e-03
FAM49A	ENSBTAG00000012779	Concordantly up	1.142	1.61e-05	1.828	8.63e-03
CD300LB	ENSBTAG00000008327	Concordantly up	4.558	1.67e-05	8.436	8.36e-05
TNFAIP3	ENSBTAG00000000436	Concordantly up	2.007	1.96e-05	4.017	2.11e-04
ADGRE5	ENSBTAG00000021818	Concordantly up	1.522	1.99e-05	1.125	4.72e-02
HHEX	ENSBTAG00000014217	Concordantly up	1.136	2.00e-05	1.587	4.90e-03
DRAM1	ENSBTAG00000017287	Concordantly up	1.144	2.02e-05	1.096	1.17e-02
SAMSN1	ENSBTAG00000002623	Concordantly up	3.105	2.08e-05	3.822	2.96e-04
TBXAS1	ENSBTAG00000020225	Concordantly up	1.154	2.13e-05	2.747	9.00e-04
RASA2	ENSBTAG00000015413	Concordantly up	1.284	2.18e-05	1.641	7.55e-03
WLS	ENSBTAG00000012976	Concordantly up	1.070	2.45e-05	1.676	1.31e-03
2005-09-01 00:00:00	ENSBTAG00000008320	Concordantly up	1.287	2.59e-05	2.225	2.97e-03
IL1A	ENSBTAG00000010349	Concordantly up	3.918	2.67e-05	6.205	3.38e-04
CCL20	ENSBTAG00000021326	Concordantly up	6.845	2.78e-05	5.124	3.51e-03
SRXN1	ENSBTAG00000010913	Concordantly up	2.056	4.28e-05	1.448	2.69e-03
MMP25	ENSBTAG00000016424	Concordantly up	1.504	4.30e-05	4.026	2.28e-03
CD69	ENSBTAG00000002135	Concordantly up	2.176	5.26e-05	7.016	1.90e-04
UBA7	ENSBTAG00000012335	Concordantly up	1.123	5.99e-05	1.631	4.91e-02
BCL3	ENSBTAG00000009493	Concordantly up	1.333	6.11e-05	2.005	4.40e-03
RND1	ENSBTAG00000018773	Concordantly up	1.598	6.82e-05	2.734	5.01e-03
PRDM1	ENSBTAG00000000816	Concordantly up	1.363	7.08e-05	1.214	4.36e-02
MCTP2	ENSBTAG00000013689	Concordantly up	2.392	7.19e-05	2.476	1.35e-02
STC1	ENSBTAG00000001687	Concordantly up	1.409	8.10e-05	2.056	2.35e-02
NR4A3	ENSBTAG00000001864	Concordantly up	3.548	1.04e-04	5.096	4.76e-04

Gene	Ensembl_ID	Class	Mastitis_log2FC	Mastitis_padj	Endometritis_log2FC	Endometritis_FDR
	ENSBTAG00000000735	Concordantly up	2.828	1.06e-04	2.770	6.92e-03
ZC3H12A	ENSBTAG00000011316	Concordantly up	1.415	1.10e-04	2.557	8.99e-04
PLCG2	ENSBTAG00000002103	Concordantly up	1.062	1.26e-04	1.289	5.03e-03
	ENSBTAG00000040392	Concordantly up	1.004	1.30e-04	2.427	1.15e-03
IL18R1	ENSBTAG00000001034	Concordantly up	3.325	1.35e-04	2.677	2.66e-03
	ENSBTAG00000009812	Concordantly up	3.555	1.36e-04	3.689	2.28e-03
HIST1H2AC	ENSBTAG00000039657	Concordantly up	1.441	1.83e-04	1.479	2.37e-02
ACTB	ENSBTAG00000026199	Concordantly up	1.042	1.92e-04	1.418	5.20e-03
CHI3L1	ENSBTAG00000018223	Concordantly up	2.923	1.92e-04	2.406	7.63e-03
BCL6	ENSBTAG00000001511	Concordantly up	1.296	2.36e-04	2.049	8.19e-04
MAP4K1	ENSBTAG00000021145	Concordantly up	1.242	2.53e-04	1.491	1.13e-02
STEAP4	ENSBTAG00000002340	Concordantly up	3.052	2.58e-04	2.203	1.73e-02
BDKRB2	ENSBTAG00000021717	Concordantly up	1.858	2.75e-04	1.227	4.19e-02
NLRC4	ENSBTAG00000008223	Concordantly up	1.709	2.91e-04	2.485	2.74e-03
LMNB1	ENSBTAG00000002882	Concordantly up	1.103	2.95e-04	3.117	2.85e-04
CEMIP	ENSBTAG00000007431	Concordantly up	3.845	3.40e-04	4.042	1.06e-04
IRF1	ENSBTAG00000031231	Concordantly up	1.035	3.49e-04	3.035	1.26e-03
	ENSBTAG00000040586	Concordantly up	5.841	3.74e-04	9.211	1.24e-04
ATG16L2	ENSBTAG00000019059	Concordantly up	1.060	3.99e-04	1.951	2.96e-04
RHOQ	ENSBTAG00000013749	Concordantly up	1.058	4.00e-04	1.056	1.18e-02
GCH1	ENSBTAG00000040151	Concordantly up	1.017	4.32e-04	2.567	8.20e-04
CD37	ENSBTAG00000011421	Concordantly up	1.171	4.43e-04	2.003	2.00e-03
IPCEF1	ENSBTAG00000003489	Concordantly up	1.737	4.97e-04	1.864	6.49e-03
SNN	ENSBTAG00000026376	Concordantly up	1.320	5.26e-04	2.217	2.49e-03
DHRS9	ENSBTAG00000004557	Concordantly up	2.951	5.26e-04	2.108	2.23e-02
	ENSBTAG00000039016	Concordantly up	5.023	5.98e-04	3.239	5.56e-04
CD274	ENSBTAG00000000095	Concordantly up	2.177	5.99e-04	4.930	6.22e-04
	ENSBTAG00000037608	Concordantly up	4.348	6.78e-04	9.573	1.12e-04
NFKBIE	ENSBTAG00000013705	Concordantly up	1.035	6.90e-04	1.671	3.82e-03

Gene	Ensembl_ID	Class	Mastitis_log2FC	Mastitis_padj	Endometritis_log2FC	Endometritis_FDR
	ENSBTAG00000006851	Concordantly up	1.316	9.16e-04	2.316	5.37e-03
GAB4	ENSBTAG00000002922	Concordantly up	1.002	1.06e-03	2.107	5.60e-03
PLAUR	ENSBTAG00000013125	Concordantly up	2.548	1.09e-03	4.281	2.34e-04
PARVG	ENSBTAG00000008057	Concordantly up	1.302	1.11e-03	1.605	2.22e-02
CD244	ENSBTAG00000002951	Concordantly up	1.110	1.15e-03	3.568	2.69e-04
SNAI1	ENSBTAG00000014554	Concordantly up	1.409	1.19e-03	2.302	8.73e-03
GLIPR1	ENSBTAG00000011590	Concordantly up	1.374	1.42e-03	1.330	1.99e-02
CASS4	ENSBTAG00000014555	Concordantly up	1.869	1.53e-03	5.892	6.48e-04
PIK3AP1	ENSBTAG00000019872	Concordantly up	1.004	1.67e-03	1.620	1.39e-02
KMO	ENSBTAG00000014439	Concordantly up	2.538	2.01e-03	2.544	2.38e-02
EIF4E3	ENSBTAG00000018862	Concordantly up	1.289	2.10e-03	1.332	1.24e-02
DNAH17	ENSBTAG00000000920	Concordantly up	1.458	2.27e-03	3.067	5.89e-04
	ENSBTAG00000047449	Concordantly up	2.671	2.32e-03	2.526	9.88e-03
MGAM	ENSBTAG00000046152	Concordantly up	3.594	2.33e-03	5.973	2.71e-04
	ENSBTAG00000009354	Concordantly up	1.054	2.38e-03	1.467	5.56e-03
TNFAIP2	ENSBTAG00000035995	Concordantly up	1.255	3.25e-03	1.313	2.04e-02
MARCKSL1	ENSBTAG00000046862	Concordantly up	1.295	3.40e-03	2.481	3.32e-03
SLC2A9	ENSBTAG00000039197	Concordantly up	1.526	3.62e-03	1.909	2.16e-03
PIK3R5	ENSBTAG00000005978	Concordantly up	1.169	4.09e-03	2.224	8.67e-03
CSF1	ENSBTAG00000000283	Concordantly up	1.006	4.90e-03	2.392	3.23e-02
AHSG	ENSBTAG00000000522	Concordantly up	1.343	4.97e-03	8.486	8.36e-05
	ENSBTAG00000039037	Concordantly up	3.937	5.98e-03	9.280	1.13e-04
	ENSBTAG00000002416	Concordantly up	1.343	6.36e-03	2.405	4.17e-03
ALPL	ENSBTAG00000008951	Concordantly up	1.257	8.55e-03	2.790	1.23e-03
CYR61	ENSBTAG00000009844	Concordantly up	1.086	9.75e-03	1.166	5.76e-03
	ENSBTAG00000039980	Concordantly up	1.399	9.75e-03	1.406	4.23e-02
AREG	ENSBTAG00000018134	Concordantly up	2.503	1.28e-02	3.967	7.53e-04
ADAMTSL4	ENSBTAG00000003604	Concordantly up	1.697	1.32e-02	1.448	5.21e-03
SLAMF1	ENSBTAG00000007927	Concordantly up	1.798	1.59e-02	2.947	2.36e-03

Gene	Ensembl_ID	Class	Mastitis_log2FC	Mastitis_padj	Endometritis_log2FC	Endometritis_FDR
ARHGAP15	ENSBTAG00000032289	Concordantly up	1.274	1.84e-02	3.249	2.80e-04
IFIT2	ENSBTAG00000034918	Concordantly up	1.125	2.20e-02	2.509	3.90e-02
NCF4	ENSBTAG00000007531	Concordantly up	1.686	2.27e-02	2.864	1.65e-03
RRAD	ENSBTAG00000013929	Concordantly up	1.511	2.36e-02	2.001	2.96e-02
LRMP	ENSBTAG00000019711	Concordantly up	1.092	3.87e-02	1.277	2.15e-02
MYBPH	ENSBTAG00000011465	Concordantly up	1.607	4.18e-02	3.880	6.76e-04
AGAP2	ENSBTAG00000007156	Concordantly up	1.056	4.31e-02	2.016	2.64e-03
SYTL3	ENSBTAG00000010334	Concordantly up	1.084	4.99e-02	4.389	3.30e-04
KRT19	ENSBTAG00000004905	Concordantly down	-2.193	2.29e-28	-2.160	1.52e-02
ATP13A4	ENSBTAG00000016305	Concordantly down	-3.199	1.96e-24	-1.716	8.90e-03
EHHADH	ENSBTAG00000019625	Concordantly down	-1.501	2.59e-18	-2.393	5.65e-04
ACSS2	ENSBTAG00000013303	Concordantly down	-2.867	1.23e-17	-1.635	1.25e-02
CYP4B1	ENSBTAG00000011976	Concordantly down	-6.110	1.35e-16	-3.764	2.54e-02
TOB1	ENSBTAG00000000546	Concordantly down	-1.811	6.12e-15	-1.226	1.21e-02
CLMN	ENSBTAG00000019916	Concordantly down	-1.748	8.11e-14	-1.847	4.59e-03
MAL2	ENSBTAG00000011779	Concordantly down	-1.507	1.03e-12	-2.333	1.06e-03
	ENSBTAG00000019093	Concordantly down	-1.619	1.38e-11	-1.640	1.42e-02
PIGR	ENSBTAG00000019798	Concordantly down	-1.599	2.19e-11	-4.129	7.98e-03
SCNN1A	ENSBTAG00000002631	Concordantly down	-2.112	5.65e-11	-2.910	4.73e-03
TPD52L1	ENSBTAG00000003336	Concordantly down	-1.737	9.11e-11	-1.574	4.39e-03
NFIX	ENSBTAG00000018229	Concordantly down	-1.308	4.64e-10	-2.198	5.13e-03
MGST1	ENSBTAG00000008541	Concordantly down	-1.179	6.65e-10	-1.035	3.09e-02
ECHDC2	ENSBTAG00000002910	Concordantly down	-1.653	6.76e-10	-1.485	8.99e-03
	ENSBTAG00000011330	Concordantly down	-1.470	9.99e-10	-1.318	4.05e-02
HES1	ENSBTAG00000000569	Concordantly down	-2.156	3.16e-09	-1.233	2.26e-03
RASAL1	ENSBTAG00000016739	Concordantly down	-1.407	4.15e-09	-1.966	1.20e-02
RHPN2	ENSBTAG00000003089	Concordantly down	-1.480	5.56e-09	-1.359	3.37e-02
IFT57	ENSBTAG00000005787	Concordantly down	-1.484	9.48e-09	-1.259	7.03e-03
CCDC170	ENSBTAG00000004099	Concordantly down	-2.900	1.04e-08	-3.140	1.33e-03

Gene	Ensembl_ID	Class	Mastitis_log2FC	Mastitis_padj	Endometritis_log2FC	Endometritis_FDR
ATP2C2	ENSBTAG00000000945	Concordantly down	-1.058	1.06e-08	-1.886	1.76e-02
SLC1A4	ENSBTAG00000007763	Concordantly down	-1.964	1.22e-08	-1.503	8.99e-03
GPAM	ENSBTAG00000011917	Concordantly down	-3.449	1.31e-08	-1.856	3.42e-04
BTC	ENSBTAG00000004237	Concordantly down	-1.113	1.37e-08	-3.010	1.90e-03
EPHX2	ENSBTAG00000015241	Concordantly down	-1.636	3.19e-08	-2.777	2.09e-03
LMO2	ENSBTAG00000006951	Concordantly down	-1.260	4.94e-08	-1.057	4.58e-02
OXTR	ENSBTAG00000019772	Concordantly down	-1.557	1.13e-07	-1.733	1.10e-02
CUEDC1	ENSBTAG00000007147	Concordantly down	-1.053	1.24e-07	-1.498	7.61e-03
PPP1R36	ENSBTAG00000007809	Concordantly down	-1.505	2.15e-07	-1.291	1.08e-02
PKP2	ENSBTAG00000002651	Concordantly down	-1.032	2.65e-07	-2.654	1.50e-03
IQGAP2	ENSBTAG00000000897	Concordantly down	-1.023	2.94e-07	-1.964	1.55e-03
NUDT12	ENSBTAG00000027728	Concordantly down	-1.088	3.28e-07	-2.293	3.01e-04
CMTM8	ENSBTAG00000001710	Concordantly down	-1.256	3.80e-07	-1.371	3.79e-02
NET1	ENSBTAG00000007648	Concordantly down	-1.095	7.27e-07	-1.635	1.83e-02
MLPH	ENSBTAG00000000634	Concordantly down	-1.867	1.87e-06	-2.267	1.21e-02
ACOX2	ENSBTAG00000004178	Concordantly down	-1.434	1.88e-06	-1.129	1.52e-02
MSRB2	ENSBTAG00000044112	Concordantly down	-1.374	2.87e-06	-1.119	5.96e-03
GIPC2	ENSBTAG00000011733	Concordantly down	-1.178	3.31e-06	-2.071	1.94e-02
PPM1K	ENSBTAG00000005754	Concordantly down	-1.587	4.57e-06	-2.361	2.11e-03
SERTAD4	ENSBTAG00000010850	Concordantly down	-1.404	5.19e-06	-1.818	1.81e-03
	ENSBTAG00000000414	Concordantly down	-1.666	1.11e-05	-2.160	2.30e-02
EFHC1	ENSBTAG00000017810	Concordantly down	-1.709	2.15e-05	-1.915	1.56e-03
KIAA1324	ENSBTAG00000001136	Concordantly down	-1.320	7.58e-05	-3.827	2.82e-03
LRIG3	ENSBTAG00000002227	Concordantly down	-1.103	1.97e-04	-2.961	1.39e-03
	ENSBTAG00000033886	Concordantly down	-1.521	3.26e-04	-1.426	3.28e-02
TFF3	ENSBTAG00000021276	Concordantly down	-1.118	5.10e-04	-5.478	1.52e-03
DUS4L	ENSBTAG00000021849	Concordantly down	-1.154	1.06e-03	-1.347	2.66e-03
NSUN7	ENSBTAG00000001572	Concordantly down	-1.003	1.35e-03	-3.513	3.01e-04
MYO1A	ENSBTAG00000014655	Concordantly down	-1.418	1.35e-03	-2.166	7.44e-03

Gene	Ensembl_ID	Class	Mastitis_log2FC	Mastitis_padj	Endometritis_log2FC	Endometritis_FDR
TMEM107	ENSBTAG00000001702	Concordantly down	-1.210	2.01e-03	-2.086	6.09e-04
C1orf168	ENSBTAG00000009314	Concordantly down	-1.796	2.42e-03	-1.900	8.45e-03
SVOPL	ENSBTAG00000031265	Concordantly down	-1.938	3.85e-03	-3.989	1.06e-03
DHRS12	ENSBTAG00000016109	Concordantly down	-1.120	4.11e-03	-1.173	2.08e-02
DZIP3	ENSBTAG00000006158	Concordantly down	-1.162	6.32e-03	-1.242	1.21e-02
ABAT	ENSBTAG00000004038	Concordantly down	-1.405	8.03e-03	-1.866	2.69e-03
TTC12	ENSBTAG00000010008	Concordantly down	-1.463	8.35e-03	-3.851	2.33e-04
SH3BGRL2	ENSBTAG00000018634	Concordantly down	-1.021	1.07e-02	-1.649	9.29e-03
OGN	ENSBTAG00000011824	Concordantly down	-1.535	2.22e-02	-2.455	6.50e-03
ANO1	ENSBTAG00000009997	Concordantly down	-1.138	2.27e-02	-2.726	2.72e-03
	ENSBTAG00000013920	Concordantly down	-1.038	4.61e-02	-4.227	1.79e-04

Table S3. Stepwise WGCNA, cis-eQTL, and machine-learning annotation of the 47 core comorbidity genes.

Gene	Core class	WGCNA_module	kME_in_module	Blue_hub(kME>0.7)	cis_eQTL_tissues	cis_eQTL_min_qval	ML_13_input	ML_raw_candidate	Final_3gene_module
IL1B	Up core	blue	0.958	Yes			No	No	No
IL6	Up core	blue	0.753	Yes	Lymph_Node; Mammary_Gland	4.01e-03	Yes	No	No
IL10	Up core	blue	0.968	Yes			No	No	No
ICAM1	Up core	blue	0.853	Yes	Mammary_Gland	7.62e-04	Yes	No	No
IL1A	Up core	Not in WGCNA network		No			No	No	No
CCL5	Up core	Not in WGCNA network		No			No	No	No
CXCL8	Up core	blue	0.893	Yes			No	No	No
TLR2	Up core	blue	0.887	Yes	Uterus	3.62e-03	Yes	No	No
CCL20	Up core	blue	0.785	Yes			No	No	No
CD40	Up core	blue	0.896	Yes	Lymph_Node	2.59e-02	Yes	No	No
CD80	Up core	blue	0.883	Yes			No	No	No
CSF1	Up core	turquoise	-0.620	No			No	No	No
ACTB	Up core	turquoise	-0.735	No			No	No	No
SELL	Up core	blue	0.850	Yes	Lymph_Node	4.60e-04	Yes	Yes	Yes
CD69	Up core	blue	0.732	Yes			No	No	No
IL1RN	Up core	blue	0.973	Yes			No	No	No
CD274	Up core	salmon	-0.868	No			No	No	No
JAK2	Up core	blue	0.916	Yes	Lymph_Node	2.55e-04	Yes	No	No
NFKBIA	Up core	blue	0.931	Yes	Mammary_Gland	1.66e-02	Yes	No	No
RELB	Up core	blue	0.917	Yes			No	No	No
PIK3CD	Up core	turquoise	-0.880	No			No	No	No
LYN	Up core	blue	0.953	Yes	Mammary_Gland	2.03e-03	Yes	No	No
SYK	Up core	blue	0.939	Yes	Lymph_Node; Mammary_Gland	5.17e-06	Yes	Yes	Yes
VAV1	Up core	turquoise	-0.915	No			No	No	No
IL6R	Up core	blue	0.895	Yes	Mammary_Gland; Uterus	1.34e-12	Yes	Yes	No
TNFAIP3	Up core	blue	0.812	Yes	Lymph_Node	5.88e-03	Yes	No	No
SELP	Up core	turquoise	-0.766	No			No	No	No
IRF1	Up core	blue	0.736	Yes	Mammary_Gland	8.19e-03	Yes	No	No
SOCS3	Up core	blue	0.958	Yes			No	No	No
ISG15	Up core	blue	0.933	Yes	Uterus	3.17e-03	Yes	Yes	Yes
ABAT	Down core	turquoise	0.881	No			No	No	No
NUDT12	Down core	turquoise	0.731	No			No	No	No
EHHADH	Down core	blue	-0.972	No			No	No	No

Gene	Core class	WGCNA_module	kME_in_module	Blue_hub(kME>0.7)	cis_eQTL_tissues	cis_eQTL_min_qval	ML_13_input	ML_raw_candidate	Final_3gene_module
ACSS2	Down core	turquoise	0.949	No			No	No	No
ACOX2	Down core	turquoise	0.867	No			No	No	No
ECHDC2	Down core	yellow	0.882	No			No	No	No
EPHX2	Down core	blue	-0.957	No			No	No	No
DUS4L	Down core	turquoise	0.765	No			No	No	No
GPAM	Down core	blue	-0.885	No			No	No	No
CYP4B1	Down core	black	-0.787	No			No	No	No
DHRS12	Down core	turquoise	0.644	No			No	No	No
ATP13A4	Down core	turquoise	0.902	No			No	No	No
MSRB2	Down core	blue	-0.883	No			No	No	No
CCDC170	Down core	turquoise	0.941	No			No	No	No
KIAA1324	Down core	Not in WGCNA network		No			No	No	No
EFHC1	Down core	blue	-0.934	No			No	No	No
CLMN	Down core	turquoise	0.954	No			No	No	No

Table S4. The 27 shared comorbidity genes contained in the preserved blue WGCNA module.

Hub genes were defined by kME > 0.7 (22 of 27).

Gene	Ensembl_ID	kME_blue	Is_hub(kME >0.7)	cis_eQTL	ML_13_input	Final_3gene_module
IL1RN	ENSBTAG00000019665	0.973	Yes	No	No	No
IL10	ENSBTAG00000006685	0.968	Yes	No	No	No
SOCS3	ENSBTAG00000008441	0.958	Yes	No	No	No
IL1B	ENSBTAG00000001321	0.958	Yes	No	No	No
LYN	ENSBTAG00000020034	0.953	Yes	Yes	Yes	No
SYK	ENSBTAG00000004767	0.939	Yes	Yes	Yes	Yes
ISG15	ENSBTAG00000014707	0.933	Yes	Yes	Yes	Yes
NFKBIA	ENSBTAG00000016683	0.931	Yes	Yes	Yes	No
RELB	ENSBTAG00000038428	0.917	Yes	No	No	No
JAK2	ENSBTAG00000012047	0.916	Yes	Yes	Yes	No
CD40	ENSBTAG00000020736	0.896	Yes	Yes	Yes	No
IL6R	ENSBTAG00000018474	0.895	Yes	Yes	Yes	No
CXCL8	ENSBTAG00000019716	0.893	Yes	No	No	No
TLR2	ENSBTAG00000008008	0.887	Yes	Yes	Yes	No
CD80	ENSBTAG00000018059	0.883	Yes	No	No	No
ICAM1	ENSBTAG00000010303	0.853	Yes	Yes	Yes	No
SELL	ENSBTAG00000011515	0.850	Yes	Yes	Yes	Yes
TNFAIP3	ENSBTAG00000000436	0.812	Yes	Yes	Yes	No
CCL20	ENSBTAG00000021326	0.785	Yes	No	No	No
IL6	ENSBTAG00000014921	0.753	Yes	Yes	Yes	No
IRF1	ENSBTAG00000031231	0.736	Yes	Yes	Yes	No
CD69	ENSBTAG00000002135	0.732	Yes	No	No	No
MSRB2	ENSBTAG00000044112	-0.883	No	No	No	No
GPAM	ENSBTAG00000011917	-0.885	No	No	No	No
EFHC1	ENSBTAG00000017810	-0.934	No	No	No	No
EPHX2	ENSBTAG00000015241	-0.957	No	No	No	No
EHHADH	ENSBTAG00000019625	-0.972	No	No	No	No

Table S5. Per-signature ssGSEA statistics and detected-marker counts in the two discovery datasets.

Dataset	Cell_type	mean_control	mean_case	delta_score	direction	p_value	FDR	significant	n_overlap_markers
GSE75379 mastitis	Neutrophils	-0.036	0.406	0.442	Ecoli_24h up	2.16e-03	1.44e-02	yes	18
GSE75379 mastitis	Macrophages	-0.060	0.162	0.222	Ecoli_24h up	2.16e-03	1.44e-02	yes	30
GSE75379 mastitis	Other immune cells	-0.071	0.241	0.312	Ecoli_24h up	2.16e-03	1.44e-02	yes	11
GSE75379 mastitis	Mast cells	-0.176	0.121	0.296	Ecoli_24h up	1.52e-02	7.58e-02	no	4
GSE75379 mastitis	CD4+ T cells	-0.320	-0.154	0.165	Ecoli_24h up	2.60e-02	8.66e-02	no	13
GSE75379 mastitis	Plasma cells	0.210	0.293	0.083	Ecoli_24h up	2.60e-02	8.66e-02	no	4
GSE75379 mastitis	Lymphatic endothelial cells	0.068	0.143	0.075	Ecoli_24h up	4.11e-02	1.18e-01	no	19
GSE75379 mastitis	Conventional dendritic cells type 1	-0.117	-0.066	0.051	Ecoli_24h up	1.32e-01	2.93e-01	no	17
GSE75379 mastitis	Luminal cells	0.401	0.433	0.033	Ecoli_24h up	1.32e-01	2.93e-01	no	12
GSE75379 mastitis	Cytotoxic CD8+ T cells	-0.081	-0.036	0.045	Ecoli_24h up	2.40e-01	4.76e-01	no	10
GSE75379 mastitis	Blood vascular endothelial cells	0.094	0.143	0.049	Ecoli_24h up	3.10e-01	4.76e-01	no	50
GSE75379 mastitis	NK cells	-0.049	0.018	0.066	Ecoli_24h up	3.10e-01	4.76e-01	no	13
GSE75379 mastitis	Glia cells	0.425	0.445	0.020	Ecoli_24h up	3.10e-01	4.76e-01	no	3
GSE75379 mastitis	T cells	-0.315	-0.258	0.058	Ecoli_24h up	3.94e-01	5.63e-01	no	11
GSE75379 mastitis	Stromal cells at intima of corpus uteri	0.102	0.149	0.048	Ecoli_24h up	4.85e-01	6.46e-01	no	10
GSE75379 mastitis	Fibroblasts	0.109	0.146	0.037	Ecoli_24h up	6.99e-01	8.23e-01	no	45
GSE75379 mastitis	Smooth muscle cells	0.313	0.333	0.021	Ecoli_24h up	6.99e-01	8.23e-01	no	18
GSE75379 mastitis	Other epithelial cells	0.466	0.471	0.005	Ecoli_24h up	8.18e-01	9.09e-01	no	14
GSE75379 mastitis	NK T cells	-0.103	-0.102	0.001	Ecoli_24h up	9.37e-01	9.87e-01	no	10
GSE75379 mastitis	WC1+ γδT cells	-0.255	-0.265	-0.010	Ecoli_24h down	1.00e+00	1.00e+00	no	6
GSE125964 endometritis	NK T cells	-0.143	0.031	0.174	Endometritis up	1.50e-03	1.92e-02	yes	10
GSE125964 endometritis	Cytotoxic CD8+ T cells	-0.072	0.139	0.211	Endometritis up	2.88e-03	1.92e-02	yes	10
GSE125964 endometritis	Blood vascular endothelial cells	0.048	0.167	0.118	Endometritis up	2.88e-03	1.92e-02	yes	50

Dataset	Cell_type	mean_control	mean_case	delta_score	direction	p_value	FDR	significant	n_overlap_markers
GSE125964 endometritis	Neutrophils	0.147	0.404	0.257	Endometritis up	5.20e-03	2.55e-02	yes	18
GSE125964 endometritis	CD4+ T cells	-0.092	0.082	0.174	Endometritis up	8.93e-03	2.55e-02	yes	13
GSE125964 endometritis	Lymphatic endothelial cells	0.043	0.166	0.123	Endometritis up	8.93e-03	2.55e-02	yes	19
GSE125964 endometritis	Other immune cells	0.202	0.394	0.192	Endometritis up	8.93e-03	2.55e-02	yes	11
GSE125964 endometritis	T cells	-0.200	-0.029	0.170	Endometritis up	1.15e-02	2.87e-02	yes	11
GSE125964 endometritis	NK cells	0.064	0.203	0.139	Endometritis up	1.47e-02	3.26e-02	yes	13
GSE125964 endometritis	Macrophages	0.172	0.383	0.211	Endometritis up	2.88e-02	5.76e-02	no	30
GSE125964 endometritis	Smooth muscle cells	0.143	0.279	0.136	Endometritis up	1.05e-01	1.91e-01	no	18
GSE125964 endometritis	WC1+ γδT cells	-0.184	-0.111	0.073	Endometritis up	1.65e-01	2.76e-01	no	6
GSE125964 endometritis	Conventional dendritic cells type 1	0.067	0.139	0.072	Endometritis up	2.80e-01	3.73e-01	no	17
GSE125964 endometritis	Mast cells	0.198	0.241	0.043	Endometritis up	2.80e-01	3.73e-01	no	4
GSE125964 endometritis	Glia cells	-0.018	0.035	0.054	Endometritis up	2.80e-01	3.73e-01	no	3
GSE125964 endometritis	Other epithelial cells	0.367	0.312	-0.055	Endometritis down	3.53e-01	4.41e-01	no	14
GSE125964 endometritis	Luminal cells	0.340	0.302	-0.038	Endometritis down	4.81e-01	5.66e-01	no	12
GSE125964 endometritis	Fibroblasts	0.162	0.305	0.143	Endometritis up	5.79e-01	6.43e-01	no	45
GSE125964 endometritis	Plasma cells	0.301	0.326	0.025	Endometritis up	6.31e-01	6.64e-01	no	4
GSE125964 endometritis	Stromal cells at intima of corpus uteri	0.307	0.316	0.010	Endometritis up	9.71e-01	9.71e-01	no	10

Table S6a. Three-algorithm consensus selection frequency across 1,000 stratified bootstrap resamples.

Gene	Attempted_bootstrap	Valid_bootstrap	Ridge_Top10_frequency	LASSO_selected_frequency	ElasticNet_Top10_frequency	Consensus_frequency	Final_3gene_module
ISG15	1000	1000	1	0.913	1	0.913	Yes
SELL	1000	1000	0.944	0.749	0.953	0.744	Yes
SYK	1000	1000	0.941	0.630	0.928	0.630	Yes
IL6R	1000	1000	0.850	0.557	0.908	0.535	raw candidate (excluded: IL6R)
ICAM1	1000	1000	0.984	0.244	0.978	0.244	No
TLR2	1000	1000	0.598	0.207	0.413	0.207	No
LYN	1000	1000	1	0.186	1	0.186	No
IRF1	1000	1000	0.627	0.061	0.719	0.057	No
NFKBIA	1000	1000	0.867	0.034	0.804	0.034	No
CD40	1000	1000	0.966	0.032	0.884	0.032	No
TNFAIP3	1000	1000	0.197	0.023	0.226	0.023	No
JAK2	1000	1000	0.973	0.021	0.942	0.021	No
IL6	1000	1000	0.053	0.001	0.245	0.001	No

Table S6b. Per-model selection count and coefficient-direction stability across 1,000 bootstrap resamples.

Model	Gene	Attempted_bootstrap	Valid_bootstrap	Selection_n	Selection_frequency	Direction_n	Positive_n	Positive_among_selected	Negative_among_selected	Positive_frequency	Negative_frequency
Ridge	ISG15	1000	1000	1000	1	1000	1000	1	0	1	0
Ridge	SELL	1000	1000	944	0.944	944	944	1	0	0.944	0
Ridge	IL6R	1000	1000	850	0.850	850	850	1	0	0.850	0
Ridge	SYK	1000	1000	941	0.941	941	941	1	0	0.941	0
LASSO	ISG15	1000	1000	913	0.913	913	913	1	0	0.913	0
LASSO	SELL	1000	1000	749	0.749	749	749	1	0	0.749	0
LASSO	IL6R	1000	1000	557	0.557	557	557	1	0	0.557	0
LASSO	SYK	1000	1000	630	0.630	630	630	1	0	0.630	0
ElasticNet	ISG15	1000	1000	1000	1	1000	1000	1	0	1	0
ElasticNet	SELL	1000	1000	953	0.953	953	953	1	0	0.953	0
ElasticNet	IL6R	1000	1000	908	0.908	908	908	1	0	0.908	0
ElasticNet	SYK	1000	1000	928	0.928	928	928	1	0	0.928	0

Table S6c. Cross-disease transfer AUC (mastitis-trained models applied to the endometritis cohort) with 95% confidence intervals.

Model	Cross_disease_transfer_AUC (GSE125964)	CI_lower	CI_upper
Ridge	0.920	0.797	1.000
ElasticNet	0.900	0.760	1.000
LASSO-LR	0.840	0.659	1.000

Table S7a. Per-gene log2 fold change, P value, and directional concordance in the two independent validation cohorts.

Cohort	Gene	Discovery_log2 FC	Validation_log2 FC	P_value	Direction_concordant
GSE24217 mastitis	ISG15	2.864	2.684	8.76e-07	TRUE
GSE24217 mastitis	SYK	1.475	0.758	4.07e-05	TRUE
GSE24217 mastitis	SELL	3.834	3.753	1.01e-09	TRUE
GSE24217 mastitis	IL6R	2.456	2.392	6.21e-08	TRUE
Xie endometritis	ISG15	3.455	2.163	1.99e-02	TRUE
Xie endometritis	SYK	1.648	NA	NA	NA
Xie endometritis	SELL	4.870	3.326	2.44e-07	TRUE
Xie endometritis	IL6R	1.009	-1.214	1.47e-02	FALSE

Table S7b. Direction-aware Stouffer Z statistics and module AUC for the four raw candidates and the final three-gene module.

Cohort	FeatureSet	Genes_used	Stouffer_Z	P_value	Module_AUC_GSE24217
GSE24217 mastitis	four_candidates	4	10.271	<2.2e-16	1
GSE24217 mastitis	final_three_module	3	8.735	<2.2e-16	1
Xie endometritis	four_candidates	3	2.916	1.77e-03	NA
Xie endometritis	final_three_module	2	5.296	5.92e-08	NA

Table S8a. Complete DGIdb 5.0 drug–gene records with approval status, interaction score, and filtering flag.

gene	drug	regulatory approval	indication	interaction score	Kept (approved & interaction_score>= 0.5)
SELL	RIVIPANSEL	Not Approved	for treatment of sickle-cell disease	13.233	No
SELL	BIMOSIAMOSE	Not Approved	antipsoriatic,antiinflammatory agent	8.822	No
SELL	RIVIPANSEL	Not Approved		4.411	No
SELL	ASELIZUMAB	Not Approved		13.233	No
ISG15	IRINOTECAN HYDROCHLORIDE	Approved	antineoplastic agent	0.778	Yes
SYK	COMPOUND 23 [PMID: 17600705]	Not Approved		0.241	No
SYK	FOSTAMATINIB DISODIUM	Approved		0.962	Yes
SYK	MIVAVOTINIB CITRATE	Not Approved		0.962	No
SYK	APITOLISIB	Not Approved		0.044	No
SYK	FOSTAMATINIB	Approved	DMARD,antiinflammatory agent	1.925	Yes
SYK	R-343	Not Approved		0.962	No
SYK	ENTOSPLETINIB	Not Approved		0.962	No
SYK	GUSACITINIB	Not Approved		0.481	No
SYK	R-348	Not Approved		0.481	No
SYK	CHEMBL:CHEMBL379975	Not Approved		0.022	No
SYK	GSK2646264	Not Approved		0.241	No
SYK	CYC-116	Not Approved		0.010	No
SYK	TAMATINIB	Not Approved		0.321	No
SYK	DASATINIB ANHYDROUS	Approved	antineoplastic agent	0.013	No
SYK	SOVLEPLENIB	Not Approved		0.962	No
SYK	R343	Not Approved	antiallergy agent	0.962	No
SYK	LANRAPLENIB	Not Approved		0.962	No
SYK	PD166285	Not Approved		0.018	No
SYK	CHEMBL:CHEMBL535331	Not Approved		0.107	No
SYK	ADAVOSERTIB	Not Approved		0.040	No
SYK	NVP-TAE684	Not Approved		0.009	No
SYK	BAY 61-3606	Not Approved		0.024	No
SYK	TAS05567	Not Approved		0.962	No
SYK	IMATINIB	Approved	antineoplastic agent	0.013	No
SYK	SYK INHIBITOR	Not Approved		0.962	No
SYK	R-333	Not Approved		0.481	No
SYK	FOSTAMATINIB DISODIUM	Not Approved		0.962	No
SYK	ERLOTINIB	Approved	antineoplastic agent	0.017	No
SYK	GUSACITINIB	Not Approved		0.192	No
SYK	PRT-2607	Not Approved		0.962	No
SYK	R-112	Not Approved		0.962	No
SYK	CENISERTIB	Not Approved		0.008	No
SYK	CERDULATINIB	Not Approved		0.241	No
SYK	SYK INHIBITOR III	Not Approved		0.107	No
SYK	GSK143	Not Approved		0.481	No

gene	drug	regulatory approval	indication	interaction score	Kept (approved & interaction_score>= 0.5)
SYK	PACLITAXEL PROTEIN-BOUND	Approved	for treatment of peripheral arterial disease (PAD),DMARD,antiinflammatory agent,antineoplastic agent	0.016	No
SYK	SP-600125	Not Approved		0.011	No
SYK	SOVLEPLENIB	Not Approved		0.962	No
SYK	LANRAPLENIB	Not Approved		0.962	No
SYK	GW441756X	Not Approved		0.015	No
SYK	TAMATINIB	Not Approved		0.048	No
SYK	CEVIDOPLENIB DIMESYLATE	Not Approved		0.962	No
SYK	P505-15	Not Approved	antiinflammatory agent	0.770	No
SYK	COMPOUND 7 [PMID: 22464456]	Not Approved		0.241	No
SYK	SYK INHIBITOR II	Not Approved		0.481	No
SYK	LANRAPLENIB	Not Approved		0.962	No
SYK	LAZERTINIB	Approved		0.192	No
SYK	MIVAVOTINIB CITRATE	Not Approved		0.962	No
SYK	CERDULATINIB	Not Approved		0.214	No
SYK	RG-1530	Not Approved		0.011	No
SYK	ER-27319	Not Approved		0.962	No
SYK	ENTRECTINIB	Approved		0.017	No
SYK	CEDIRANIB	Not Approved	antineoplastic agent	0.020	No
SYK	MIVAVOTINIB	Not Approved		0.192	No
SYK	ILORASERTIB	Not Approved		0.009	No

Table S8b. Retained HERB 2.0 literature-supported traditional Chinese medicine candidates.

Core gene	Traditional Chinese medicine	Latin name / Formula	Evidence type	Reference
SYK	Dictamni Cortex	*Dictamni Cortex (root bark)*	Published experimental/literature only (inferred excluded)	[33]
SYK	processed Aconiti Radix	*Aconiti Radix Cocta*	Published experimental/literature only (inferred excluded)	[34]
ISG15	Yu-Ping-Feng San	Yu-Ping-Feng San (formula)	Published experimental/literature only (inferred excluded)	[35]
SELL	Ononis spinosa	*Ononis spinosa L. (root)*	Published experimental/literature only (inferred excluded)	[36]
